# Synthesis of Meso-Substituted Subphthalocyanine–Subporphyrin Hybrids: Boron Subtribenzodiazaporphyrins**

**DOI:** 10.1002/anie.201502662

**Published:** 2015-05-15

**Authors:** Sonia Remiro-Buenamañana, Alejandro Díaz-Moscoso, David L Hughes, Manfred Bochmann, Graham J Tizzard, Simon J Coles, Andrew N Cammidge

**Affiliations:** School of Chemistry, University of East Anglia Norwich Research Park, Norwich NR4 7TJ (UK) E-mail: a.cammidge@uea.ac.uk; UK National Crystallography Service, School of Chemistry, University of Southampton Southampton SO17 1BJ (UK)

**Keywords:** dyes/pigments, heterocycles, phthalocyanines, porphyrinoids, synthetic methods

## Abstract

The first syntheses of hybrid structures that lie between subphthalocyanines and subporphyrins are reported. The versatile single-step synthetic method uses a preformed aminoisoindolene to provide the bridging methine unit and its substituent while trialkoxyborates simultaneously act as Lewis acid, template, and provider of the apical substituent. The selection of each component therefore allows for the controlled formation of diverse, differentially functionalized systems. The new hybrids are isolated as robust, pure materials that display intense absorption and emission in the mid-visible region. The new compounds are further characterized in solution and solid state by variable-temperature NMR spectroscopy and X-ray crystallography, respectively.

Macrocyclic oligopyrrole structures are ubiquitous in both nature and everyday life. The general class is exemplified by the two symmetric frameworks of porphyrin **1** (4 pyrrole units linked by methine bridges) and phthalocyanine **2** (4 isoindole units linked by nitrogen bridges; Figure 1). Many thousands of studies have been published covering their design, synthesis, properties, and applications, and research effort continues to increase.[1]

**Figure 1:**
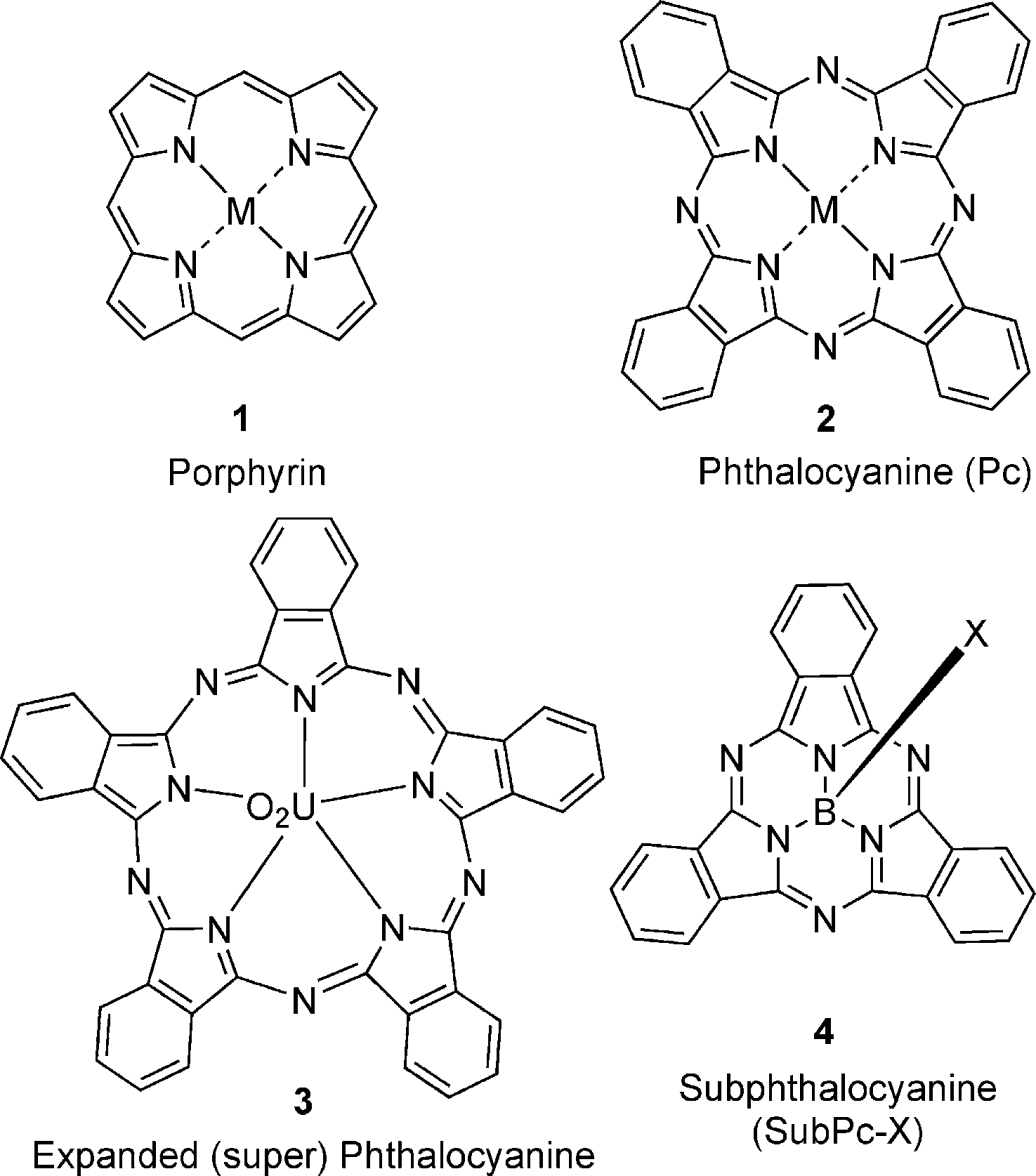
The parent structures porphyrin 1, phthalocyanine 2, expanded phthalocyanine 3, and subphthalocyanine 4 (M=metal or H, H).

Of particular importance are recent synthetic efforts to prepare modified structures where the macrocyclic core (usually aromatic) itself is perturbed, yielding derivatives with fundamentally different properties and uses.[2–5] Studies have focused separately on ring-expanded[6] and -contracted[7] analogues. The manipulation of porphyrin-like structures has proved most straightforward for ring-expanded systems, with synthetic strategies building on established polypyrrole construction methods. Expanded phthalocyanine structures include the uranyl derivative **3**,[8] which comprises five indole-type units. Ring-contracted phthalocyanines[7] are especially intriguing and have been the subject of growing research efforts since the serendipitous formation of boron subphthalocyanine (SubPc, **4**) by Meller and Ossko in 1972.[9]

SubPcs[7, 10] adopt a bowl-like conformation. The central boron atom bears a further apical group (X in **4**), which can be interchanged under appropriate conditions.[7] The optical properties of SubPcs are distinctive as they show intense absorption and emission around 550 nm, leading to a particular focus on their application as components in photovoltaic cells.[7, 11–13] A small number of subporphyrins are also known, the most notable examples being the synthetic breakthroughs reported by Osuka and Kobayashi leading to the first syntheses of boron tribenzosubporphyrins **5**[14] and **6**[15] (Figure 2).

**Figure 2:**
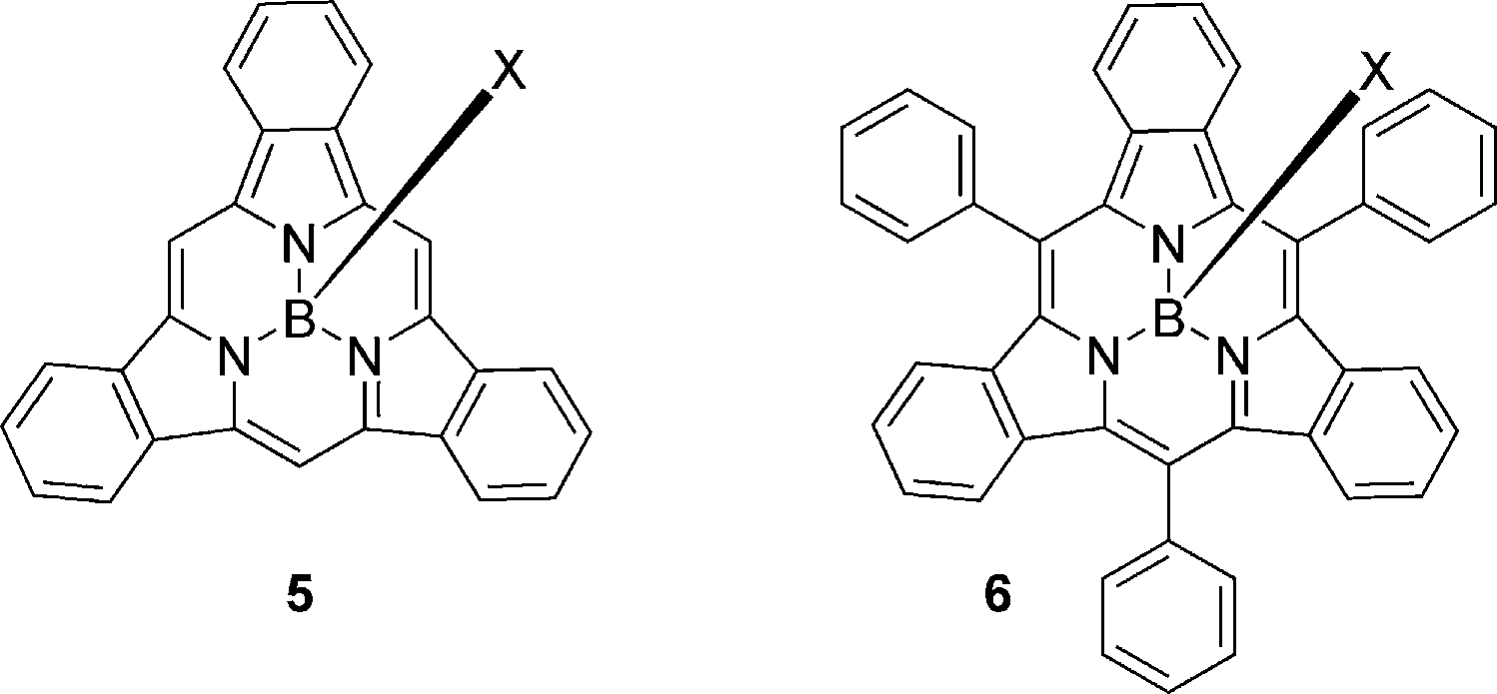
Tribenzosubporphyrins reported by the Osuka (5) and Kobayashi (6) groups.

The most challenging core modification of the oligopyrrole/indole macrocycles is arguably the generation of hybrid structures[16] that lie between the classic porphyrin and phthalocyanine parent structures. The challenge posed by these systems is perhaps best illustrated by the limited number of studies to date,[16] despite their first identification being reported in the late 1930s by Dent[17] and the Linstead group.[18] The greatest drawback to the investigation of the hybrid structures is the limited synthetic possibilities available to access modified and bespoke derivatives. Nevertheless, interest in the hybrid structures has accelerated rapidly over the last decade even though most syntheses have relied on the original procedures, albeit with some improvements.[16, 19, 20] We recently reported a significant breakthrough in this area,[21] disclosing a new, versatile procedure that gives controlled access to functionalized TBTAPs (TBTAP=tetrabenzotriazaporphyrin, a phthalocyanine–tetrabenzoporphyrin hybrid in which a single aza bridge of the Pc ring is replaced by carbon). Importantly, the synthesis allows for the introduction of substituents at the “new” meso site. Cheprakov and co-workers[22] have subsequently reported a complementary approach to a second member of the hybrid series, the *trans*-TBDAPs (Scheme 1). Until now, hybrid structures based on the ring-contracted systems, SubPc and tribenzoSubPn, are unknown. Herein, we report a versatile method that provides access to the first examples of such hybrids, alongside a preliminary examination of their properties.

**Scheme 1:**
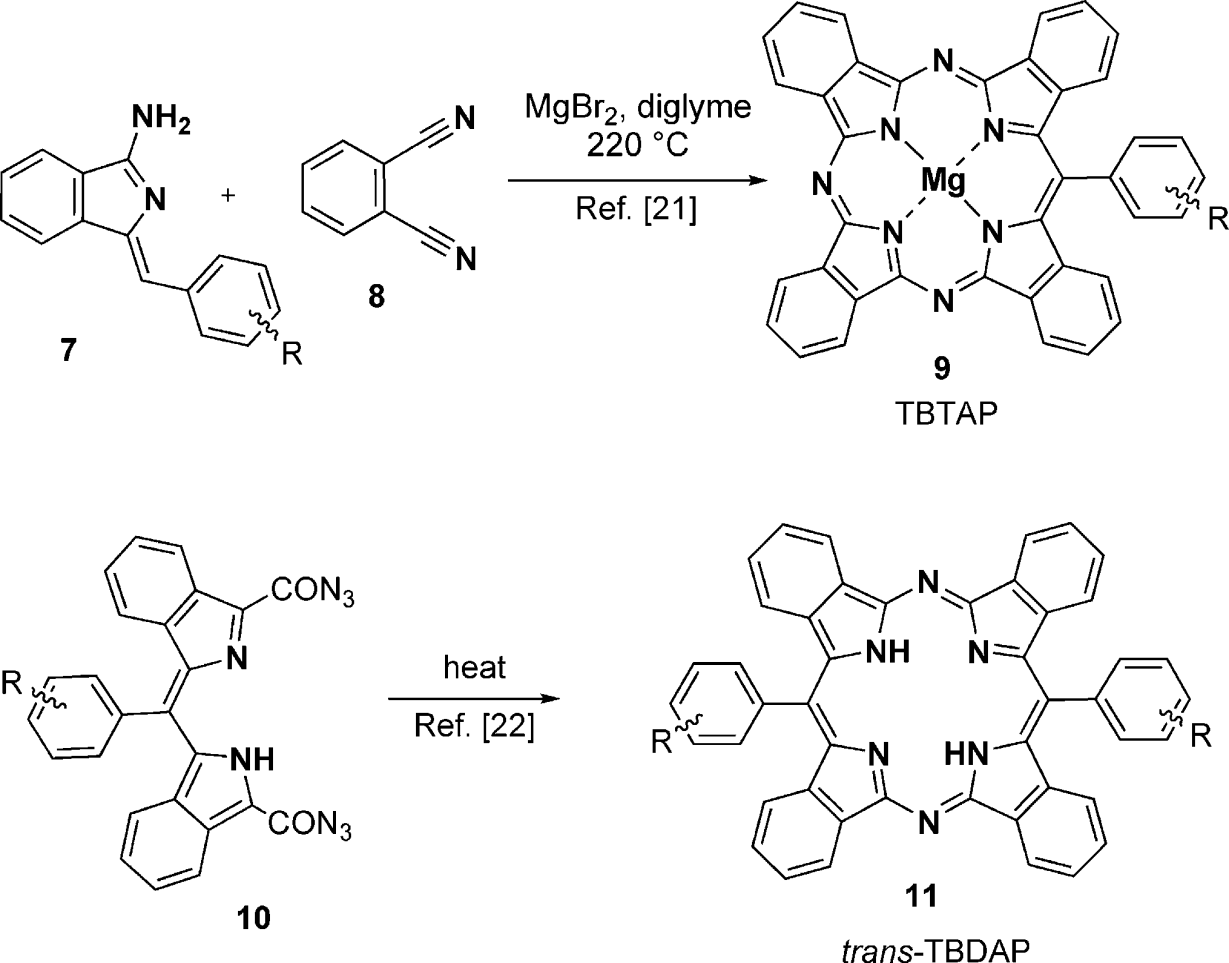
Synthetic breakthroughs giving straightforward access to functionalized porphyrin–phthalocyanine hybrids.

The optimized synthesis of SubPc **4** (X=Cl) typically involves the reaction of phthalonitrile (**8**) with BCl_3_ at elevated temperature (xylene at reflux).[7, 23] In these reactions, the boron reagent acts as a Lewis acid and as a template. In our recently developed synthesis of TBTAP hybrids **9**, we employed aminoisoindolene precursor **7** to initiate the macrocyclization process around a magnesium template ion, and we therefore reasoned that the same precursor could potentially initiate macrocyclization in the presence of a boron template leading to a hybrid in which a (functionalized) meso carbon atom formally replaces one of the bridging nitrogen atoms present in the SubPc parent. The target product is a boron subtribenzodiazaporphyrin (SubTBDAP). Consequently, in the initial set of successful reactions, aminoisoindolene **12**[24] was reacted with phthalonitrile in the presence of BCl_3_ in xylene at reflux. Three distinct, highly colored products were formed. The first product was identified as the simple, symmetric SubPc-Cl, formed by the expected homomacrocyclization of phthalonitrile. MALDI-MS studies strongly implied that the other two new compounds were the required SubTBDAP-Cl and the azaBODIPY-type compound formed from self-condensation[25] of the aminoisoindolene precursor **12** followed by complexation with boron. However, attempts to isolate and characterize the new products were impeded by the reactivity of the labile B–Cl bond present in all components of the mixture. Therefore, in a subsequent reaction (Scheme 2), macrocyclization was performed again, but followed directly by treatment of the crude product mixture with an excess amount of phenol to convert all B–Cl fragments into stable B–OPh moieties. Separation of the mixture could then be achieved by chromatography, allowing for the isolation of the first pure SubTBDAP **13** as a bright pink material with intense yellow fluorescence (*ϕ*≈0.5, Stokes shift ca. 15 nm). The yield (15 %) is impressive, especially when compared to typical yields of both simple SubPcs and unsymmetric phthalocyanines. The absorption and emission spectra are compared to those of the parent SubPc (**4**, X=OPh) in Figure 3. The absorbance and fluorescence maxima are both blue-shifted by approximately 10–20 nm compared to those of SubPc, reflecting the change towards a more porphyrin-like character in the hybrid **13**. The absorption spectrum of SubTBDAP also shows the main band to be split—a consequence of the reduced symmetry in the macrocyclic chromophore core. The blue shift also indicates that as expected, the appended meso phenyl substituent does not contribute to the main π-system, lying essentially perpendicular to the SubTBDAP core. This parallels the arrangement in both the well-known meso-phenyl porphyrins and the meso-aryl TBTAPs.

**Figure 3:**
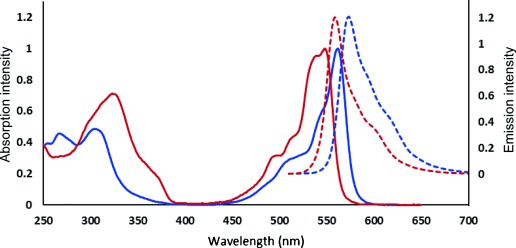
Absorption (—) and emission (– – –) spectra of hybrid SubTBDAP 13 (red) and SubPc-OPh (blue).

**Scheme 2:**
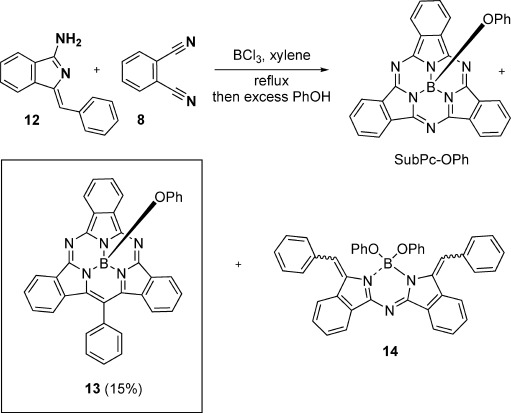
Synthesis of a boron subphthalocyanine–subporphyrin hybrid, a so-called SubTBDAP (13).

The ^1^H NMR spectrum for hybrid **13** at room temperature shows the expected signals for the macrocyclic core and the apical O–Ph group (signals for the O–Ph group are shielded by the core ring current). However, the signals for the *ortho-* and *meta-*hydrogen atoms of the meso phenyl substituent are very broad and featureless, indicating slow rotation of the group. Lowering the temperature leads to sharpening and resolution of the signals (Figure 4). A large chemical shift difference is observed for the two doublets corresponding to the *ortho*-hydrogen atoms, and they were assigned based on their sensitivity to interchange of the apical alkoxide substituent. H_*o*_, which lies under the conical macrocycle, appears at approximately 6.9 ppm in all cases, whereas the resonance for H_*o*′_, the proton lying on the same side as the alkoxide, is sensitive to its nature and is seen in the range of 8.2–8.6 ppm. The assignment was further supported by calculations (see the Supporting Information). Crystals suitable for X-ray diffraction were eventually grown from a mixture of acetone and hexane allowing for the determination of the solid-state structure.[26] In the crystal, the boron atom is bonded to the three pyrrole nitrogen atoms and the phenoxy oxygen atom (which is in the apical site of the pyramidal arrangement) in a tetrahedral (or trigonal pyramidal) arrangement (Figure 4). The boron atom is displaced 0.614(5) Å from the plane of the three N atoms, and the SubTBDAP ring system is wrapped around the base; each benzene ring plane is tilted by approximately 20° with respect to the plane of the three pyrrole N atoms away from the phenoxy group. The meso phenyl group was confirmed to be lying essentially perpendicular to the SubTBDAP core.

**Figure 4:**
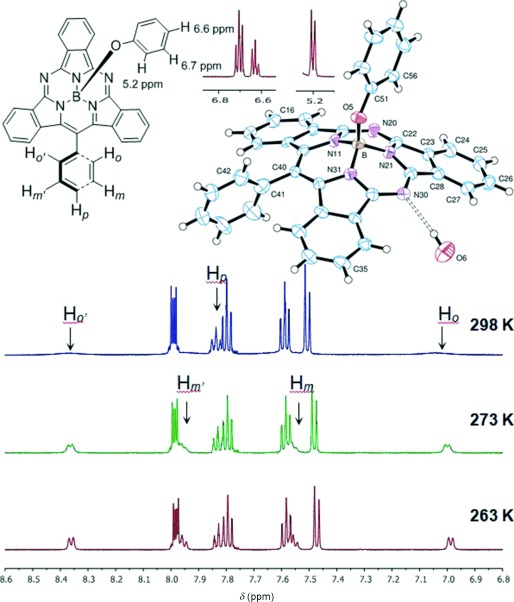
Variable-temperature ^1^H NMR spectra of hybrid 13 and its crystal structure.

The SubTBDAP molecules are arranged in pairs with the overlapping, centrosymmetrically related N21/C29 isoindole rings approximately 3.62 Å apart; these groups are also bridged by a pair of solvent water molecules, which appear to form hydrogen bonds to the nitrogen atoms N20 and N30.

The synthesis outlined above allowed for the isolation of a SubTBDAP hybrid. However, the versatility of the procedure suffers from the practical drawback of the simultaneous formation of a symmetric SubPc. The formation of the SubPc is of course expected because these initial conditions are known to lead to the macrocyclization of phthalonitrile alone. We therefore turned our attention to the development of reaction conditions that would lead to the formation of the hybrid structures (macrocyclization initiated by aminoisoindolenes **7**) without competing formation of the SubPc. This was achieved by employing less reactive boronate esters as the Lewis acid/template, recognizing that phthalonitrile itself requires a more reactive boron source for SubPc formation. The use of boronate esters (B(OR)_3_) has the added advantage of leading directly to the stable SubTBDAP-OR variants in a single operation (Scheme 3). Diglyme was found to be a convenient and suitable solvent, and the reaction between the aminoisoindolene precursor and phthalonitrile (1:3) proceeded smoothly at 200 °C in three hours. SubPc was not formed, and the hybrid SubTBDAPs were easily isolated. Yields were marginally lower than for the stepwise procedure (typically around 10 %) but compensated by synthetic convenience and versatility. Excess phthalonitrile can be recovered from the reaction alongside the hydrolysis products (phthalimide). The main side products are the expected azadipyrromethene (from self-condensation) and polar/oligomeric baseline material. The reactions can also be performed in xylene or indeed in the absence of solvent.

**Scheme 3:**
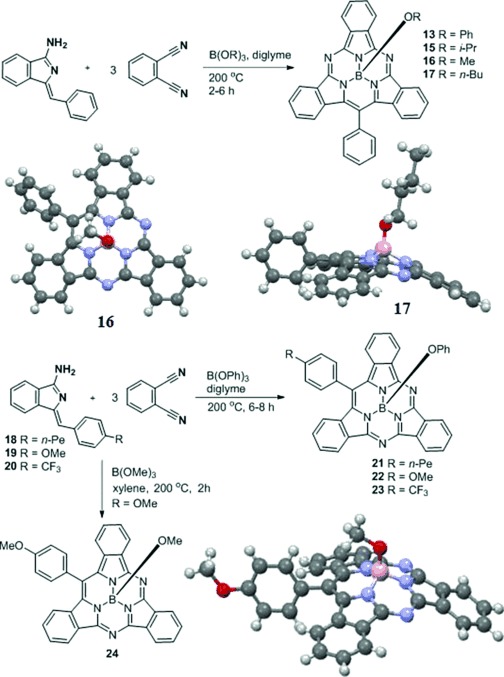
Versatile syntheses of TBDAP hybrids enabling the selective functionalization at the meso aryl and apical sites.

The scope of the method has been demonstrated by the derivatives prepared to date (Scheme 3). The selection of a boronate ester template leads to control over the apical substituent (**13**, **15**–**17**). Most significant, however, is the ability to engineer the new “meso” substituents at will—synthetic control that makes this new class of hybrid structures uniquely attractive. Aminoisoindolenes **7** are easily prepared from acetylenic precursors,[24] and the macrocyclization reaction tolerates the manipulation of this group, which was exemplified by the formation of **21**–**23**, examples of hybrid SubTBDAPs bearing alternative aryl substituents. All of the products were isolated as pure, freely soluble, crystalline solids. Crystal structures have been obtained for **15**–**17** (see the Supporting Information for that of **15**) and also for **24** (Scheme 3).[26]

In conclusion, we have reported the first examples of a new class of hybrid macrocyclic systems whose core structure lies between those of subphthalocyanines and subporphyrins. The synthesis is controlled, allowing for the manipulation of the functional groups at both the central boron atom and importantly at the new meso site. Of particular significance is the synthetic ease and versatility that will permit imaginative design of bespoke materials to exploit the intrinsic potential of the intriguing core structure.

## Supporting Information

As a service to our authors and readers, this journal provides supporting information supplied by the authors. Such materials are peer reviewed and may be re-organized for online delivery, but are not copy-edited or typeset. Technical support issues arising from supporting information (other than missing files) should be addressed to the authors.

miscellaneous_information
