# Supplementary material for: Synthesis of Meso-Substituted Subphthalocyanine–Subporphyrin Hybrids: Boron Subtribenzodiazaporphyrins
Source: Angew Chem Int Ed Engl. 2015 May 15;54(26):7510–4. doi: 10.1002/anie.201502662 (PMC4643193; doi:10.1002/anie.201502662)
Supplement: Supplementary file 1 — miscellaneous_information [file anie0054-7510-sd1.pdf]

Angewandte Chemie
Eine Zeitschrift der Gesellschaft Deutscher Chemiker

Supporting Information

Synthesis of Meso-Substituted Subphthalocyanine–Subporphyrin Hybrids: Boron Subtribenzodiazaporphyrins**

*Sonia Remiro-Buenamañana, Alejandro Díaz-Moscoso, David L. Hughes, Manfred Bochmann, Graham J. Tizzard, Simon J. Coles, and Andrew N. Cammidge**

anie_201502662_sm_miscellaneous_information.pdf

Supporting Information

Contents

General methods……………………………………………………………………………………………….2

Compound synthesis and characterisation spectra…………………………………………….3

X-Ray crystallography data……………………………………………………………………………..25

General methods:

Reagents and solvents were purchased from commercial sources and used without further purification, with the following exceptions: phthalonitrile was recrystallized from hot xylene; recrystallizations were performed using distilled solvents. ^{1}H, ^{13}C, ^{11}B, ^{19}F-NMR spectra were recorded at 500.1, 125.7, 160.5 and 470.4 MHz, respectively, using a Bruker AscendTM 500. The residual solvent peaks were used as references in the case of ^{1}H and ^{13}C, and the instrument calibrated with external references such as $BF_3{\cdot}OEt_2$ for ^{11}B-NMR and $CFCl_3$ for ^{19}F-NMR.

Spectra of SubTBDAPs are of recrystallized samples (distilled dichloromethane/distilled petroleum ether). 2D COSY, NOESY and ROESY experiments were used to assist with the NMR spectroscopy assignments. Microwave reactions used a Biotage Initiator 2.5 reactor (400W) equipped with an external temperature sensor. Thin layer chromatography (TLC) was carried out on aluminium sheets coated with silica gel 60 F252 (Merck), with visualization by UV light and by charring with 0.1% ninhydrin in EtOH when necessary. Column chromatography was carried out on silica gel Davisil® LC60A 40-63 micron (Grace GmbH & Co). MALDI-TOF mass spectra were obtained using a Shimadzu Biotech Axima instrument. High resolution mass spectrometry was performed by the ESPRC UK National Mass Spectrometry Service Centre at Swansea. UV-Vis and emission spectra were recorded at room temperature on a Hitachi U-3000 spectrophotometer and on a Hitachi F-4500 fluorescence spectrophotometer, respectively. Melting points were measured using a Reichert Thermovar microscope with a thermopar based temperature control. X-Ray crystallography data was collected and analysed by Dr Simon J. Coles and Dr Graham J. Tizzard at the UK National Crystallography Service at Southampton and by Dr David Hughes at UEA. NMR calculations (supporting assignment of H*o* and H*o*') were computed at the BP86-D3/def2-SVP level of theory. The calculations have been performed using the program TURBOMOLE version 6.4.[1] For the calculations we have used the BP86[2] functional.

Fluorescence quantum yield:

The fluorescence quantum yield (Φ_F) values of SubTBDAPs were measured in DCM, due to good solubility of the compounds in this solvent. The measurements were performed under aerobic conditions. The quantum yields were determined using Rhodamine B in MeOH as fluorescence standard;[3] it presents similar UV-Vis absorption and fluorescence maxima to the new compounds. λ_{exc} = 500 nm was chosen to obtain the emission spectra of each compound, since both standard and unknown compounds show similar absorbance at this wavelength. The Φ_F values were calculated using equation 1:

$$\Phi_u = \Phi_s \left(\frac{IA_u}{IA_s}\right)\left(\frac{\eta_u^2}{\eta_s^2}\right)$$

Where Φ is the quantum yield, IA the integrated area under the emission curve, η the refractive index of the solvents, and *u* and *s* are the subscripts used to refer to the unknown and the standard, respectively.

Aminoisoindolenes 12,[4] 18[5] and 19[6] were prepared as previously described in literature.

(Z)-1-(4-Trifluoromethylphenylmethylene)-1H-isoindol-3-amine (20)

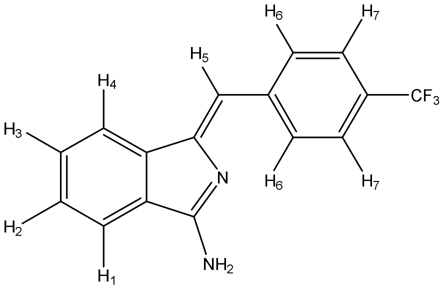

Following the reported procedure[4] a mixture of *o*-bromobenzamidine hydrochloride[7] (707.3 mg, 3.003 mmol, 1eq), BINAP (106.1 mg, 0.165 mmol, 0.055 eq) and $PdCl_2(MeCN)_2$ (39.3 mg, 0.150 mmol, 0.05 eq) was sealed in a microwave vessel with a magnetic stirrer bar and then purged and refilled with N_2 three times. Then, a solution of 1-ethynyl-4-(trifluoromethyl)benzene (0.6 mL, 3.6 mmol, 1.2 eq) and DBU (1.14 mL, 7.51 mmol, 2.5 eq) in dry DMF (12 mL) was added. The mixture was stirred under N_2 for 5 min. Finally, the mixture was irradiated in a microwave reactor at 120 °C for 1 h. After cooling, 50 mL of ethyl acetate were added and the mixture washed with a saturated solution of $NaHCO_3$ (75 mL) three times. The organic layer was dried over $MgSO_4$, filtered and concentrated. The residue was finally purified by column chromatography using 100% dichloromethane to 1:1 ethyl acetate/petroleum ether to 100% ethyl acetate as solvent gradient. The resulting solid was recrystallized from a 1:1 mixture of dichloromethane/petroleum ether to yield the *title compound* as yellow crystals (635.5 mg, 73%).

Rf 0.32 Hexane/ THF/MeOH (10:3:1)

Mp 161 - 162 °C

^{1}H-NMR (500.1 MHz, $CDCl_3$, 298 K): δ (ppm) = 8.20 (d, 2H, J = 8.1 Hz, H_6), 7.80 (d, 1H, J = 7.6 Hz, H_4), 7.61 (d, 2H, J = 8.1 Hz, H_7), 7.53 – 7.44 (m, 2H, H_1, H_3), 7.41 (t, 1H, J = 7.1 Hz, H_2), 6.73 (s, 1H, H_5).

^{13}C-NMR (125.7 MHz, $CDCl_3$, 298 K): δ (ppm) = 166.0, 149.6, 142.9, 140.4, 131.4, 130.5, 129.7, 128.6 (q, 1C, ^2J = 32.3 Hz), 127.9, 125.4 (q, 2C, ^3J = 3.8 Hz), 124.5 (q, 1C, ^1J = 272 Hz), 120.1, 119.2, 113.5.

^{19}F-NMR (470.6 MHz, $CDCl_3$, 298 K): δ (ppm) = -62.41 (s, 1F, -C$\underline{F}_3$).

MS (MALDI-TOF): *m/z* = 288.93 $[M]^+$ (100%)

HR-MS (FTMS + p NSI) ($C_{16}H_{12}N_3F_2$) $[M+H]^+$: Calc.: 289.0947; Found: 289.0946

UV-Vis (CH_2Cl_2): λ max (nm) (ε ($dm^3·mol^{-1}·cm^{-1}$)) = 364 ($2.45·10^4$), 284 ($9.32·10^3$).

^{1}H-NMR (500.1 MHz, $CDCl_3$, 298 K) Expansion

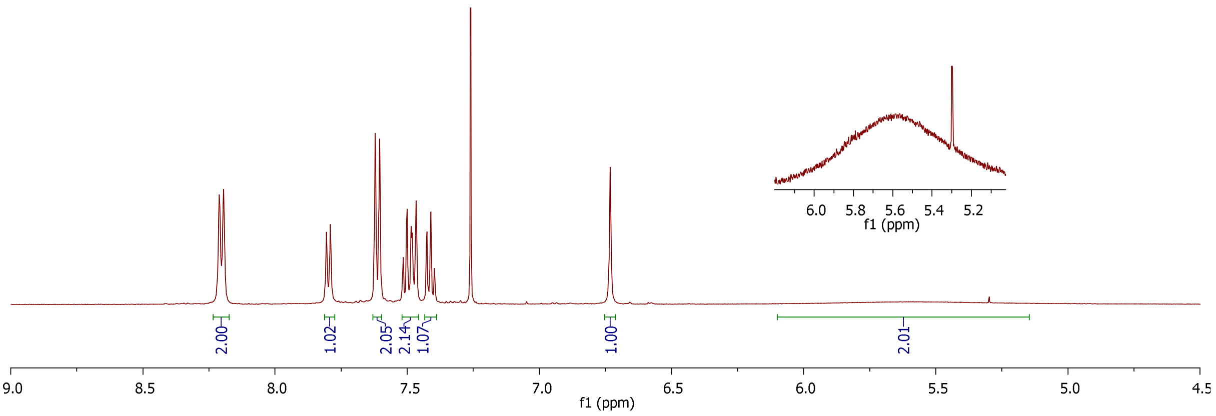

^{13}C-NMR (125.7 MHz, $CDCl_3$, 298 K)

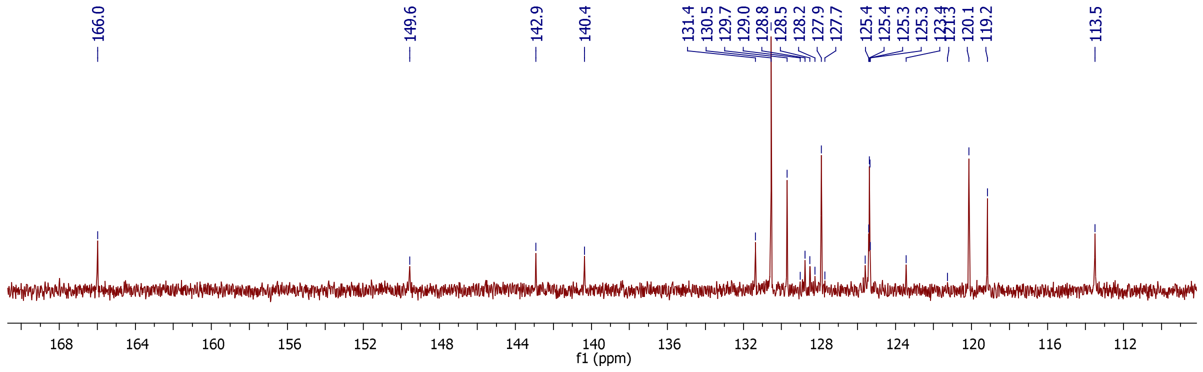

^{19}F-NMR (470.6 MHz, $CDCl_3$, 298 K)

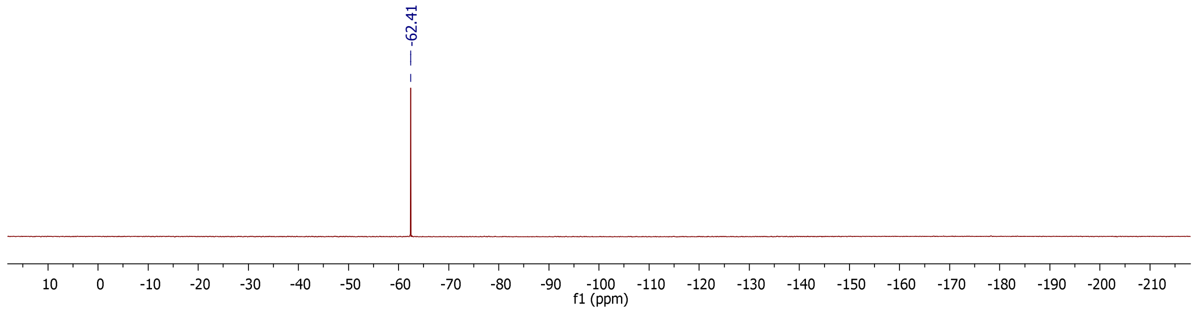

***meso*-PhenylSubTBDAP-OPh (13)**

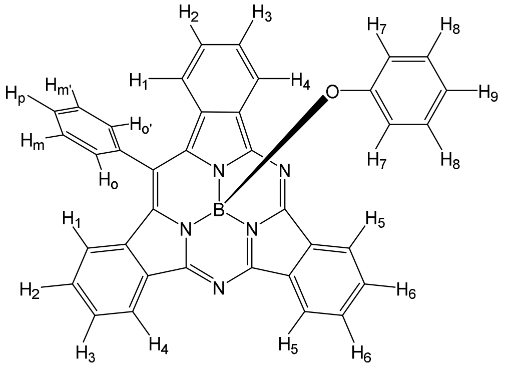

Procedure A.

(Z)-1-(Phenylmethylene)-1H-isoindol-3-amine **12**[4] (51.3 mg, 0.233 mmol, 1 eq.) and phthalonitrile (59.7 mg, 0.466 mmol, 2 eq.) were dissolved in 3 mL of *p*-xylene under argon. BCl_3 (1M in *p*-xylene, 0.47 mL, 0.47 mmol, 2 eq.) was added dropwise. The mixture was refluxed under argon for 3 hours, after which an excess of phenol (219 mg, 2.33 mmol, 10 eq) was added and the reaction was refluxed for further 19 hours. The solvent was evaporated and the crude residue was subjected to silica gel column chromatography using 2:7 ethyl acetate/hexane as eluent. The intensely yellow fluorescent product was easily monitored on the column by irradiating with a 366 nm UV lamp. A second silica gel column chromatographic separation (eluent 1:3:2 diethyl ether/hexane/dichloromethane) and recrystallization from distilled DCM/distilled petroleum ether yielded the pure *title compound* as pink-red solid (19.5 mg, 15%). A single crystal suitable for X-ray analysis was prepared by recrystallization from a mixture of acetone/hexane.

Procedure B.

A mixture of (Z)-1-(phenylmethylene)-1H-isoindol-3-amine **12**[4] (99.0 mg, 0.449 mmol, 1 eq.), phthalonitrile (173 mg, 1.35 mmol, 3 eq.) and triphenyl borate (263 mg, 0.898 mmol, 2 eq.) where dissolved in diglyme (3.3 mL), degassed with argon and placed in a sealed tube. The reaction was heated to 200 ^{o}C for 3 hours, after which time tlc indicated complete consumption of **12**. After cooling, ethyl acetate (50 mL) was added and the mixture washed with a saturated solution of $NaHCO_3$ (75 mL x 3). The organic layer was dried over $MgSO_4$, filtered and concentrated. The mixture was purified as above to yield the *title compound* as pink-red crystals (25.0 mg, 10%).

Rf 0.47 Ethyl Acetate/ Cyclohexane (2:7)

Mp 329 °C

^{1}H-NMR (500.1 MHz, CD_2Cl_2, 263 K): δ (ppm) = 8.93 – 8.90 (m, 2H, H_5), 8.89 (br d, *J* = 8.0 Hz, 2H, H_4), 8.36 (d, *J* = 7.6 Hz, 1H, $H_{o'}$), 8.00 – 7.96 (m, 2H, H_6), 7.95 (t, *J* = 7.6 Hz, 1H, $H_{m'}$), 7.83 (tt, *J* = 7.6, 1.2 Hz, 1H, H_p), 7.79 (ddd, *J* = 8.0, 7.0, 1.0 Hz, 2H, H_3), 7.58 (ddd, *J*=8.0, 7.0, 1.0 Hz, 2H, H_2), 7.56 (t, *J* = 7.6 Hz, 1H, H_m), 7.47 (d, *J* = 8.0 Hz, 2H, H_1), 6.99 (d, *J* = 7.6 Hz, 1H, H_o), 6.70 (t, *J* = 7.4 Hz, 2H, H_8), 6.63 (t, *J* = 7.4 Hz, 1H, H_9), 5.25 (d, *J* = 7.4 Hz, 2H, H_7).

^{13}C-NMR (125.7 MHz, CD_2Cl_2, 298 K): δ (ppm) = 153.1, 150.3, 150.1, 135.4, 134.1, 132.6, 131.5, 130.0, 129.9, 129.6, 129.2, 128.8, 128.6, 127.9, 127.8, 124.4, 123.0, 122.7, 122.2, 121.5, 119.7.

^{11}B-NMR (160.5 MHz, $CDCl_3$, 298 K): δ (ppm) = -14.68 (s, 1B).

MS (MALDI-TOF): *m/z* = 563.41 $[M]^+$ (100%)

HR-MS (FTMS + p APCI) ($C_{37}H_{23}B_1N_5O_1$) $[M+H]^+$: Calc.: 564.1997; Found: 564.1976

UV-Vis (distilled CH_2Cl_2): λ max (nm) (ε ($dm^3 \cdot mol^{-1} \cdot cm^{-1}$)) = 548 ($5.57 \cdot 10^4$), 324 ($3.96 \cdot 10^4$).

Fluorescence (CH_2Cl_2, Excitation at 500 nm): 559 nm, Φ_F= 0.44

^{1}H-NMR (500.1 MHz, CD_2Cl_2, 263 K)

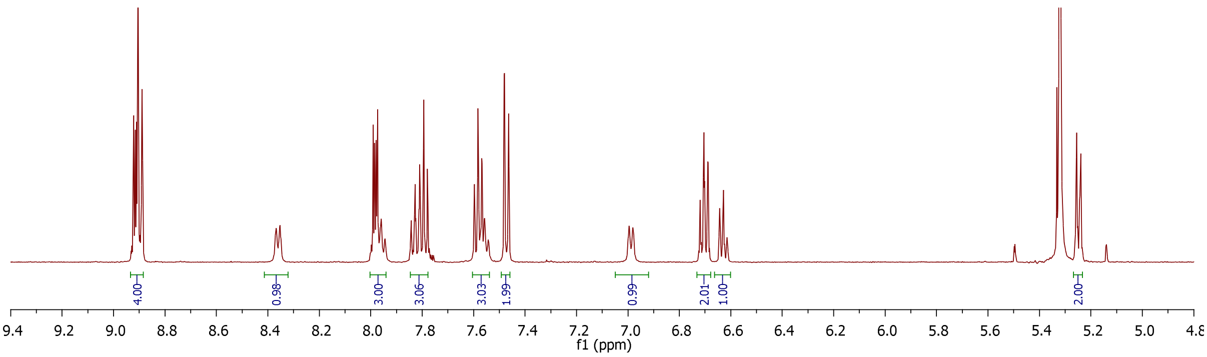

^{13}C-NMR (125.7 MHz, CD_2Cl_2, 298 K)

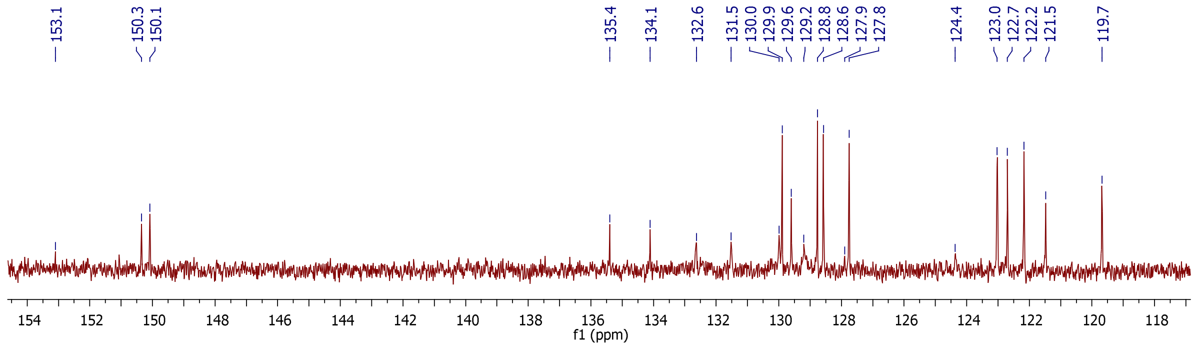

^{11}B-NMR (160.5 MHz, $CDCl_3$, 298 K)

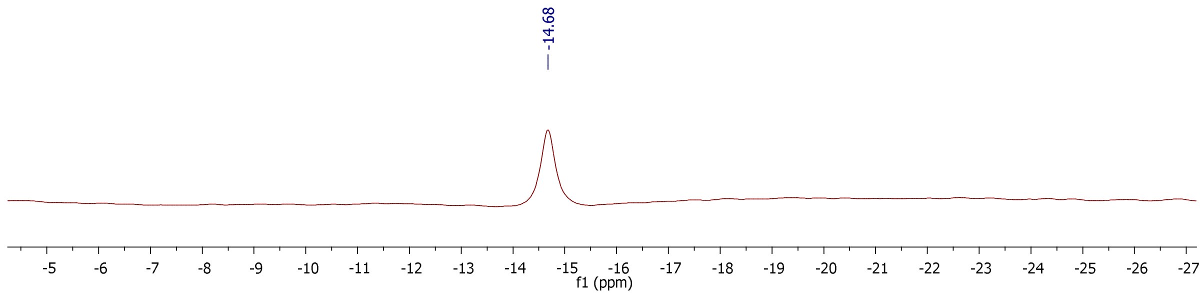

Variable temperature COSY (500.1 MHz, CD_2Cl_2, 263 K)

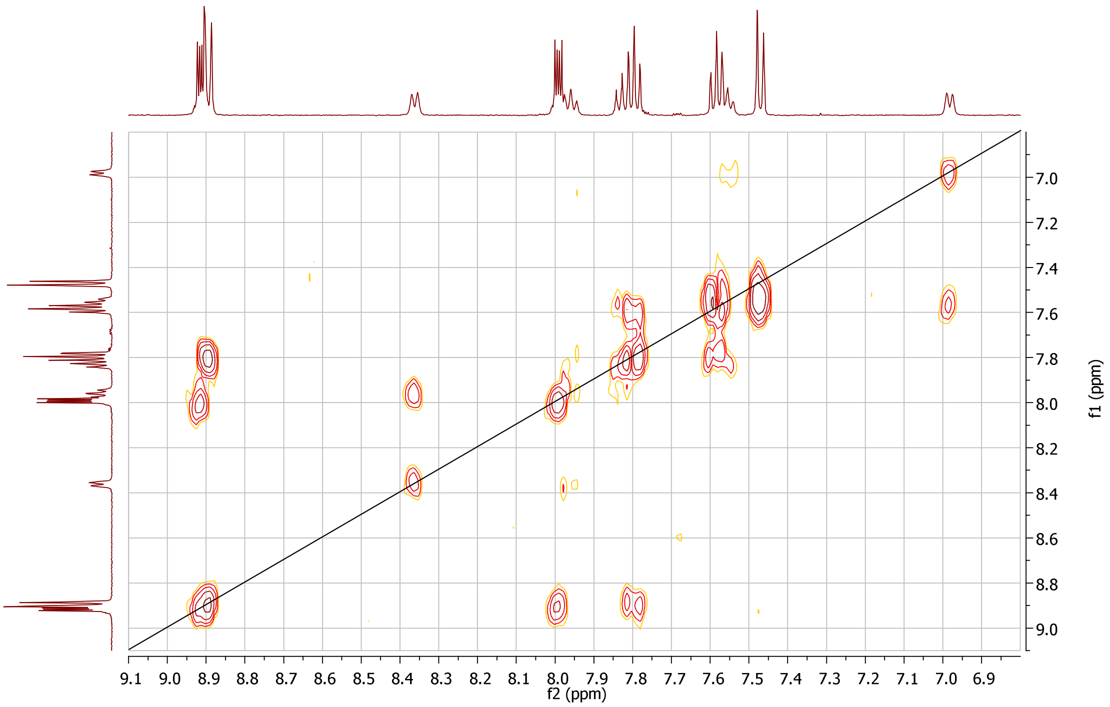

Variable temperature NOESY (500.1 MHz, CD_2Cl_2, 263 K)

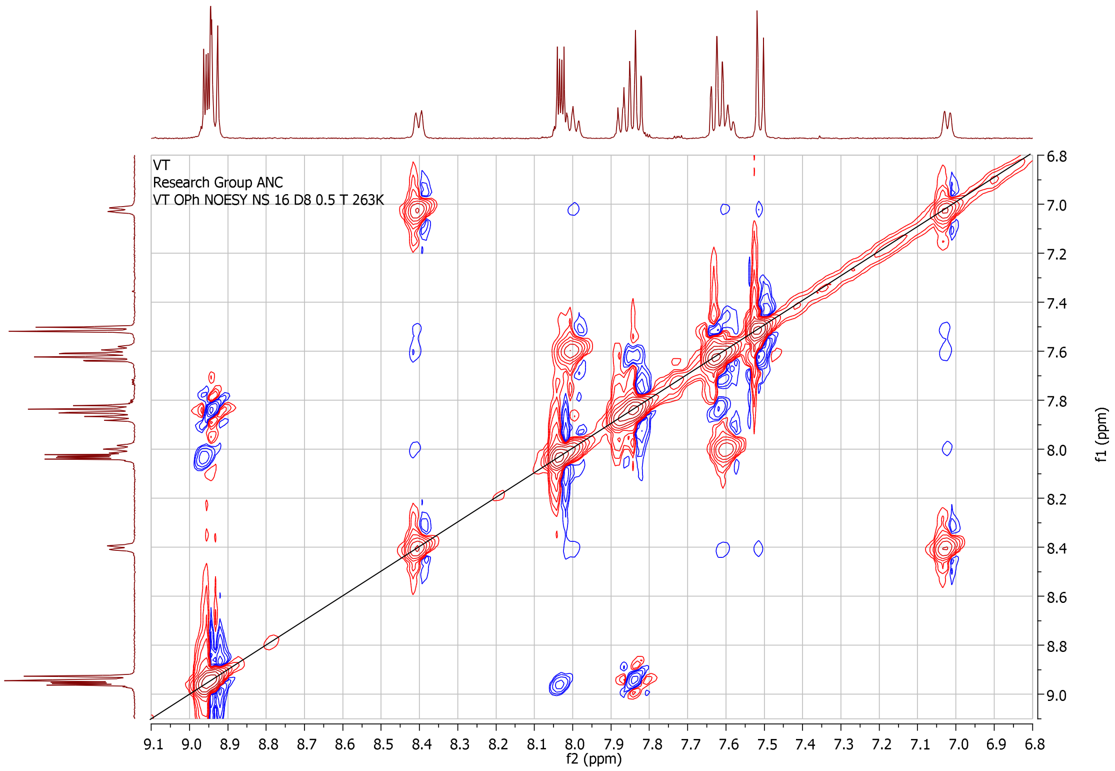

AzaBODIPY-OPh (14)

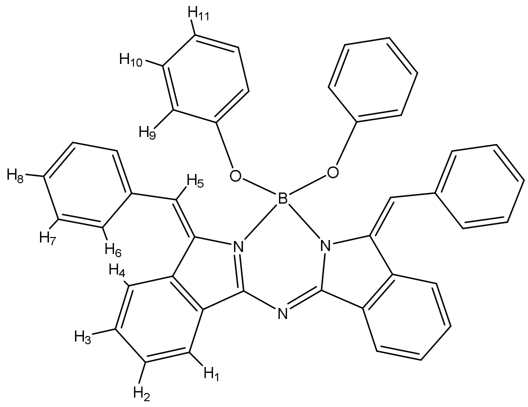

Rf 0.56 Ethyl Acetate/ Cyclohexane (2:7)

Mp 180 – 182 °C

^{1}H-NMR (500.1 MHz, CD_2Cl_2, 263 K): δ (ppm) = 8.36 (s, 2H, H_5), 8.15 (d, 2H, J = 7.6 Hz, H_1), 7.54 – 7.37 (m, 16H, H_2, H_3, H_4, H_6, H_7, H_8), 7.02 (t, 4H, J = 8.0 Hz, H_{10}), 6.79 – 6.72 (m, 6H, H_9, H_{11}).

^{13}C-NMR (125.7 MHz, CD_2Cl_2, 298 K): δ (ppm) = 164.5, 156.8, 138.4, 136.2, 135.3, 133.0, 132.3, 129.7, 129.4, 129.2, 128.7 (4), 128.7 (0), 127.5, 123.7, 123.6, 120.5, 119.3.

^{11}B-NMR (160.5 MHz, $CDCl_3$, 298 K): δ (ppm) = 2.34 (s, 1B).

MS (MALDI-TOF): m/z = 620.68 $[M+H]^+$ (100%)

HR-MS (FTMS + p NSI) ($C_{42}H_{30}B_1N_3O_2$) $[M+H]^+$: Calc.: 620.2511; Found: 620.2502

UV-Vis (distilled CH_2Cl_2): λ max (nm) (ε ($dm^3 \cdot mol^{-1} \cdot cm^{-1}$)) = 451 ($1.82 \cdot 10^4$), 319 ($2.05 \cdot 10^4$).

Fluorescence (distilled CH_2Cl_2, Excitation at 450 nm): 548 nm

^{1}H-NMR (500.1 MHz, $CDCl_3$, 298 K)

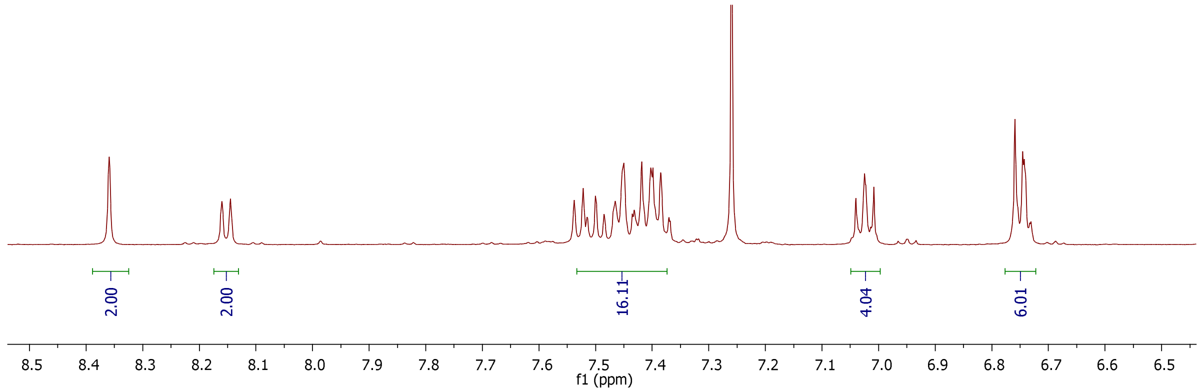

^{13}C-NMR (125.7 MHz, $CDCl_3$, 298 K)

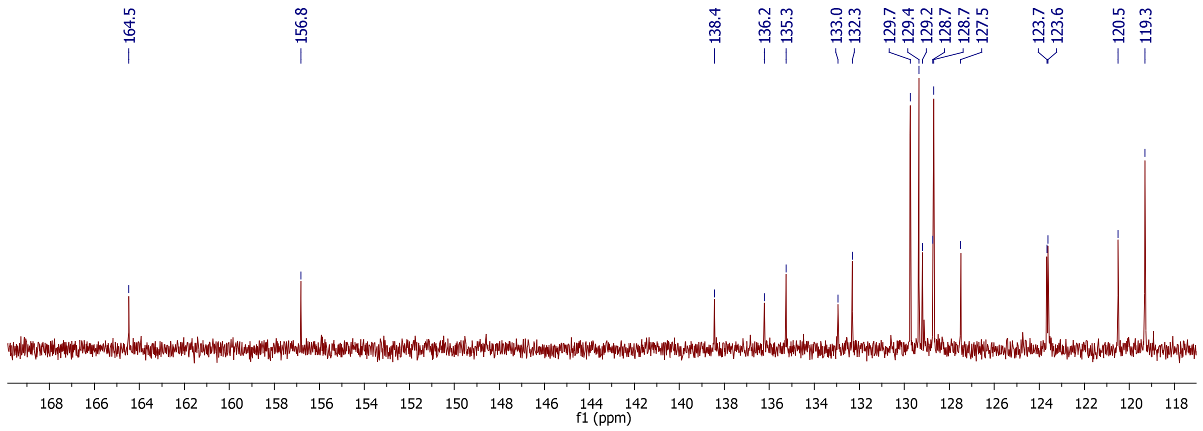

^{11}B-NMR (160.5 MHz, $CDCl_3$, 298 K)

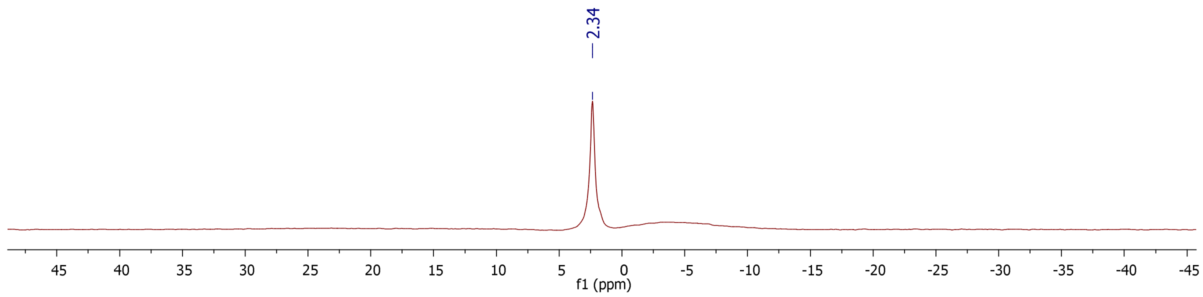

Hybrids –13-24 were prepared following Procedure B using isoindolenes 12, 18-20 and the appropriate boronate ester.

Compound	*Reaction time*	*1st column*	*2nd column*	*Yield*
**15**	6 h	1:4:2 Et_2O / Hexane /DCM	none	23.3mg, 9.2 %
**16**	4 h	1:4:2 Et_2O / Hexane /DCM	none	13.7 mg, 9 %
**17**	2 h	100% DCM -> 1:4:2 Et_2O/Hex/DCM	2:7 Ethyl Acetate /Hexane	11.8 mg, 7.4 %
**21**	6 h	1:4:2 Et_2O / Hexane /DCM	100% Hex -> (1:1) Hex/DCM -> 100% DCM	6.5 mg, 11 %
**22**	8 h	1:3:2 Et_2O / Hexane /DCM	2:7 Ethyl Acetate /Hexane	13.2 mg, 12 %
**23**	6 h	2:7 Ethyl Acetate /Hexane	1:1 Hex/ DCM -> 100% DCM	17.5 mg, 6.8 %
**24**	2 h*	1:4:2 Et_2O / Hexane /DCM	2:7 Ethyl Acetate /Hexane	16.6 mg, 10.3 %

*Reaction solvent: *p*-xylene

***meso*-PhenylSubTBDAP-O^{i}Pr (15)**

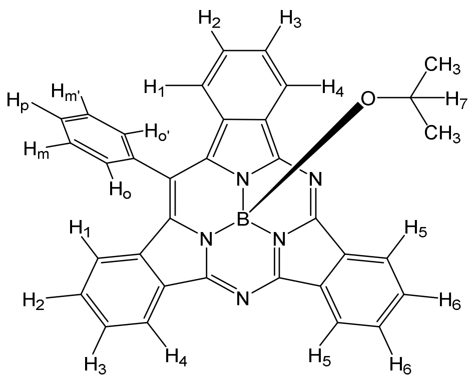

Rf 0.44 Ethyl Acetate/ Cyclohexane (2:7)

Mp 322 °C

^{1}H-NMR (500.1 MHz, CD_2Cl_2, 263 K): δ (ppm) = 8.92 – 8.89 (m, 2H, H_5), 8.88 (br d, 2H, J = 8.0 Hz, H_4), 8.57 (d, 1H, J = 7.6 Hz, $H_{o'}$), 8.01 – 7.95 (m, 3H, $H_{m'}$, H_6), 7.83 (t, 1H, J = 7.6 Hz, H_p), 7.77 (br t, 2H, J = 7.4 Hz, H_3), 7.59 – 7.53 (m, 3H, H_m, H_2), 7.47 (br d, 2H, J = 8.0 Hz, H_1), 6.96 (d, 1H, J = 7.6 Hz, H_o), 1.30 – 1.24 (m, 1H, H_7), -0.13 (d, J = 6.1 Hz, 6H, -C$\underline{H}_3$).

^{13}C-NMR (125.7 MHz, CD_2Cl_2, 298 K): δ (ppm) = 151.0, 150.6, 136.0, 134.8, 134.2, 133.0, 131.9, 130.4, 130.2, 130.0, 129.6, 128.8, 128.0, 124.9, 123.4, 122.8, 122.2, 61.7, 24.4.

^{11}B-NMR (160.5 MHz, $CDCl_3$, 298 K): δ (ppm) = -14.85 (s, 1B).

MS (MALDI-TOF): *m/z* = 529.39 $[M]^+$ (100%)

HR-MS (FTMS + p APCI) ($C_{34}H_{25}B_1N_5O_1$) $[M+H]^+$: Calc.: 530.2153; Found: 530.2143

UV-Vis (CH_2Cl_2): λ max (nm) (ε ($dm^3 \cdot mol^{-1} \cdot cm^{-1}$)) = 547 ($5.19 \cdot 10^4$), 325 ($3.98 \cdot 10^4$).

Fluorescence (CH_2Cl_2, Excitation at 500 nm): 558 nm, Φ_F= 0.43.

^{1}H-NMR (500.1 MHz, CD_2Cl_2, 263 K)

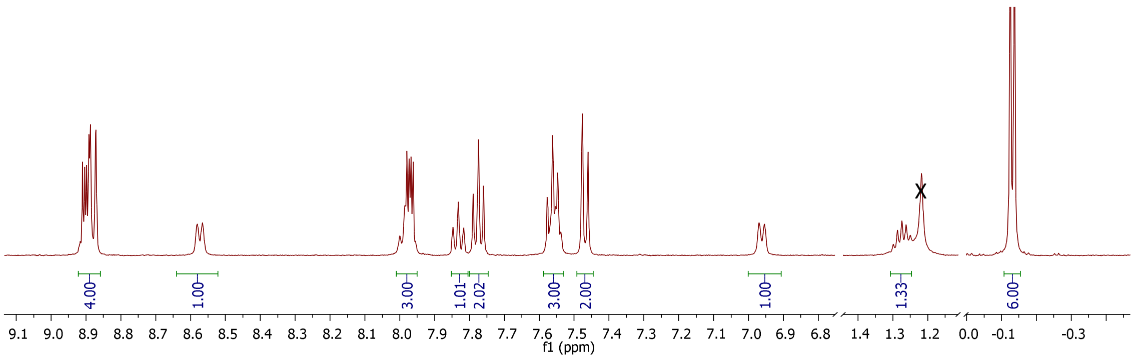

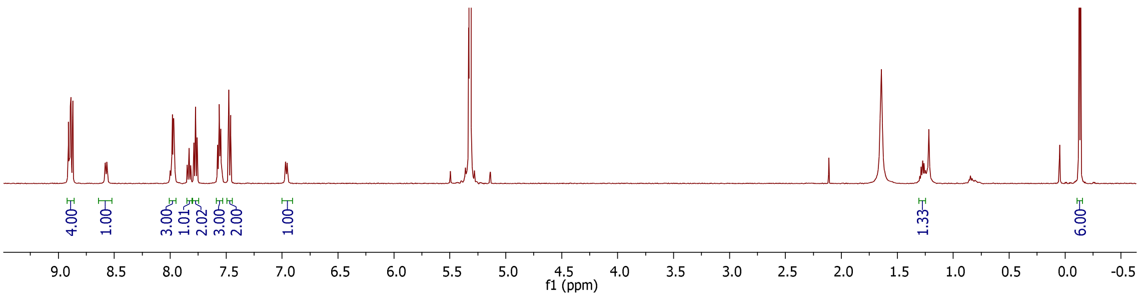

^{13}C-NMR (125.7 MHz, CD_2Cl_2, 298 K)

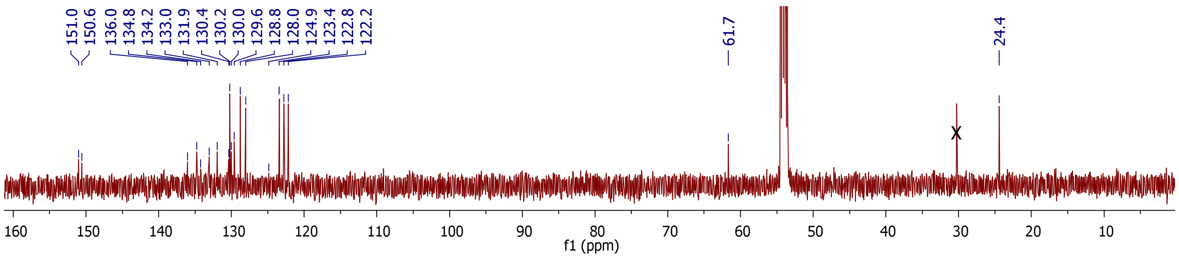

^{11}B-NMR (160.5 MHz, $CDCl_3$, 298 K)

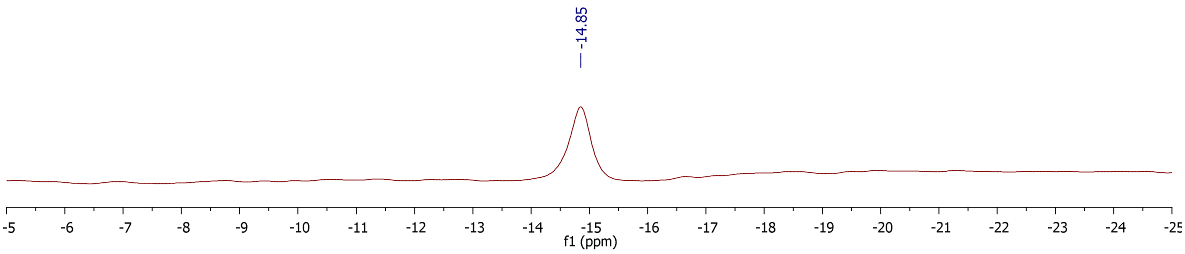

***meso*-PhenylSubTBDAP-OMe (16)**

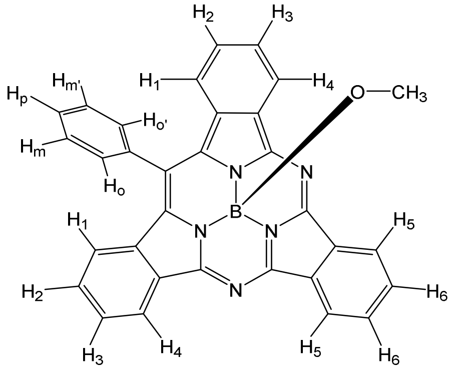

Rf 0.32 Ethyl Acetate/ Cyclohexane (2:7)

Mp 319 °C

^{1}H-NMR (500.1 MHz, CD_2Cl_2, 263 K): δ (ppm) = 8.92 – 8.88 (m, 4H, H_4, H_5), 8.58 (d, 1H, *J* = 7.6 Hz, $H_{o'}$), 7.99 (t, 1H, *J* = 7.6 Hz, $H_{m'}$), 7.97 – 7.93 (m, 2H, H_6), 7.83 (tt, 1H, *J* = 7.6, 1.2 Hz, H_p), 7.78 (ddd, 2H, *J* = 8.0, 7.1, 0.9, H_3), 7.59 – 7.53 (m, 3H, H_m, H_2), 7.47 (br d, 2H, *J* = 8.0 Hz, H_1), 6.96 (d, 1H, *J* = 7.6 Hz, H_o), 1.28 (s, 3H, -$C\underline{H}_3$).

^{13}C-NMR (125.7 MHz, CD_2Cl_2, 298 K): δ (ppm) = 151.2, 150.7, 136.0, 135.0, 134.8, 133.0, 131.9, 130.3, 130.3, 130.0, 129.6, 128.8, 128.1, 124.9, 123.4, 122.8, 122.2, 47.0.

^{11}B-NMR (160.5 MHz, $CDCl_3$, 298 K): δ (ppm) = -14.45 (s, 1B).

MS (MALDI-TOF): *m/z* = 501.27 $[M]^+$ (100%)

HR-MS (FTMS + p APCI) ($C_{32}H_{21}B_1N_5O_1$) $[M+H]^+$: Calc.: 502.1839; Found: 502.1820

UV-Vis (CH_2Cl_2): λ max (nm) (ε ($dm^3 \cdot mol^{-1} \cdot cm^{-1}$)) = 547 ($5.97 \cdot 10^4$), 324 ($4.61 \cdot 10^4$).

Fluorescence (CH_2Cl_2, Excitation at 500 nm): 558 nm, Φ_F= 0.43

^{1}H-NMR (500.1 MHz, CD_2Cl_2, 263 K)

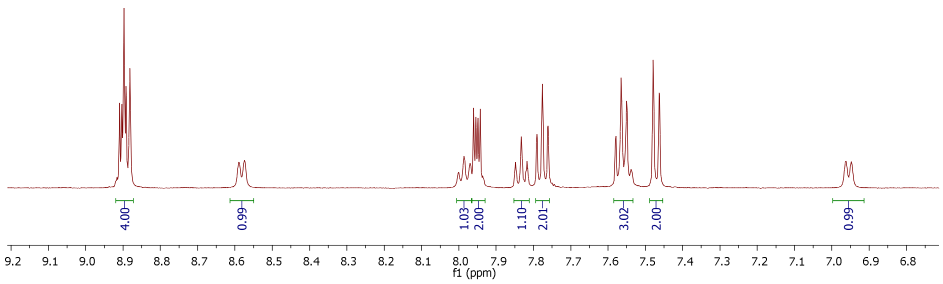

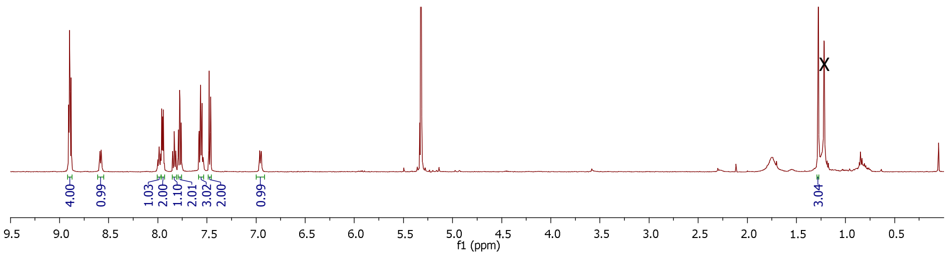

^{13}C-NMR (125.7 MHz, CD_2Cl_2, 298 K)

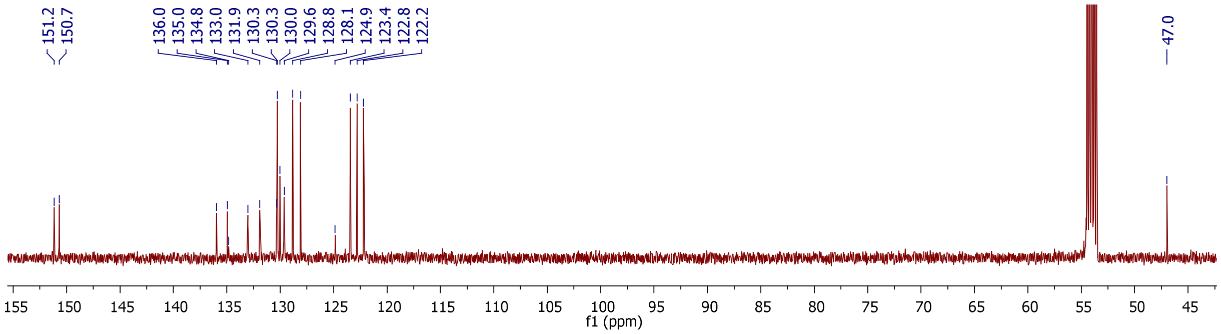

^{11}B-NMR (160.5 MHz, $CDCl_3$, 298 K)

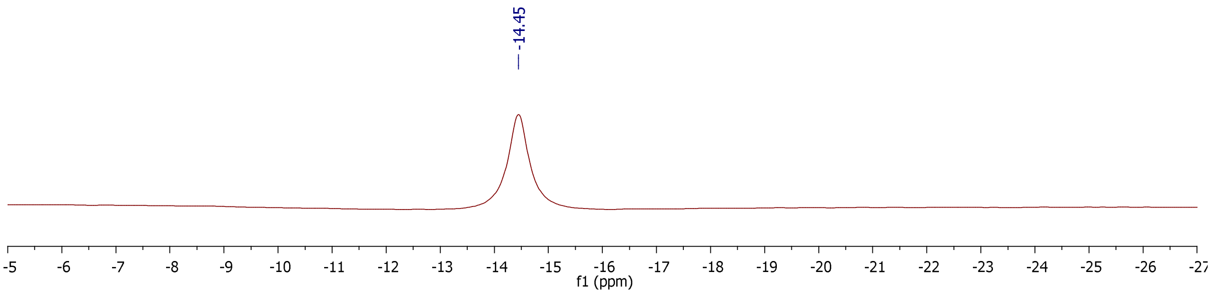

meso-PhenylSubTBDAP-OBu (17)

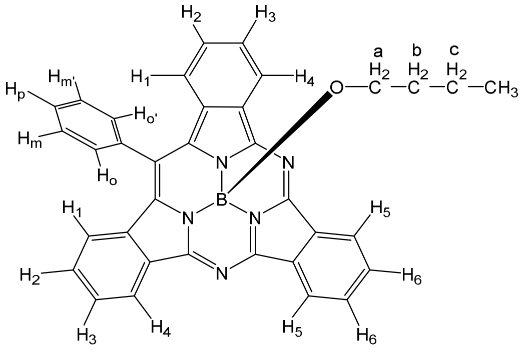

Rf 0.47 Ethyl Acetate/ Cyclohexane (2:7)

Mp 295 °C

^{1}H-NMR (500.1 MHz, CD_2Cl_2, 298 K): δ (ppm) = 8.96 – 8.91 (m, 4H, H_4, H_5), 8.57 (d, 1H, *J* = 7.6 Hz, $H_{o'}$), 8.01 – 7.99 (m, 3H, $H_{m'}$, H_6), 7.84 (t, 1H, *J* = 7.6 Hz, H_p), 7.79 (ddd, 2H, *J* = 8.0, 7.0, 1.0 Hz, H_3), 7.59 – 7.54 (m, 3H, H_m, H_2), 7.47 (br d, 2H, *J* = 8.0 Hz, H_1), 6.96 (d, 1H, , *J* = 7.6 Hz, H_o), 1.28 (t, 2H, *J* = 6.6 Hz, -$C\underline{H}_2$ a), 0.49 – 0.39 (m, 2H, $C\underline{H}_2$ b), 0.37 – 0.30 (m, 5H, -$C\underline{H}_2$ c, -$C\underline{H}_3$).

^{13}C-NMR (125.7 MHz, CD_2Cl_2, 298 K): δ (ppm) = 151.1, 150.6, 136.0, 134.9, 133.0, 131.9, 130.3, 130.2, 130.0, 129.6, 128.8, 128.1, 124.8, 123.4, 122.8, 122.2, 59.2, 33.4, 18.9, 13.7.

^{11}B-NMR (160.5 MHz, CD_2Cl_2, 298 K): δ (ppm) = -14.66 (s, 1B).

MS (MALDI-TOF): *m/z* = 543.24 $[M]^+$ (100%)

HR-MS (FTMS + p NSI) ($C_{35}H_{27}B_1N_5O_1$) $[M+H]^+$: Calc.: 544.2309; Found: 544.2297

UV-Vis (CH_2Cl_2): λ max (nm) (ε ($dm^3{\cdot}mol^{-1}{\cdot}cm^{-1}$)) = 547 ($4.85{\cdot}10^4$), 325 ($3.74{\cdot}10^4$).

Fluorescence (CH_2Cl_2, Excitation at 500 nm): 558 nm, Φ_F= 0.45

^{1}H-NMR (500.1 MHz, CD_2Cl_2, 263 K)

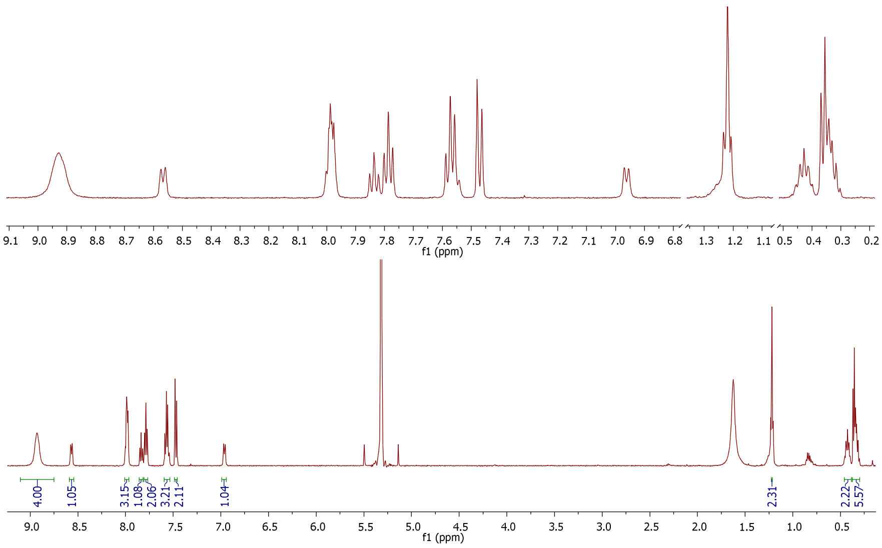

^{1}H-NMR Variable temperature experiment comparison (500.1 MHz, CD_2Cl_2)

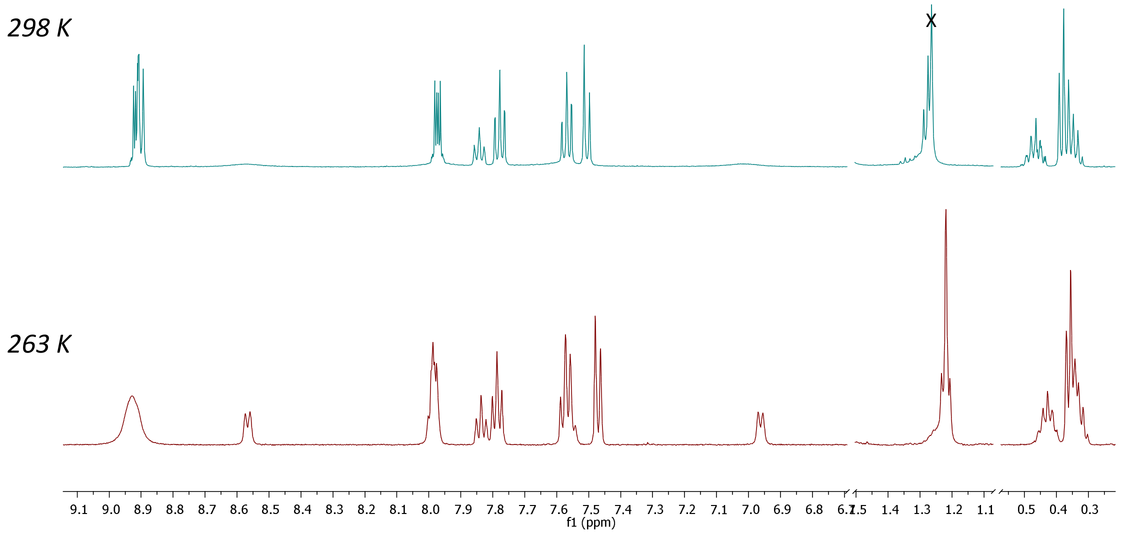

^{13}C-NMR (125.7 MHz, CD_2Cl_2, 298 K)

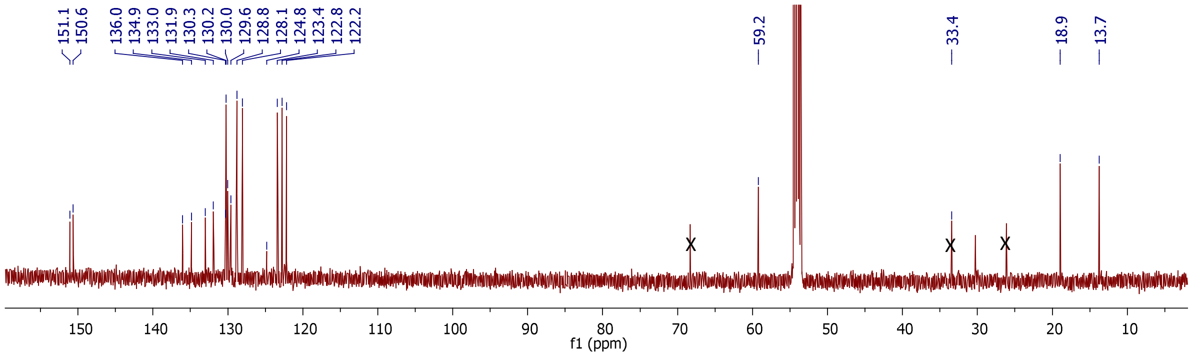

^{11}B-NMR (160.5 MHz, CD_2Cl_2, 298 K)

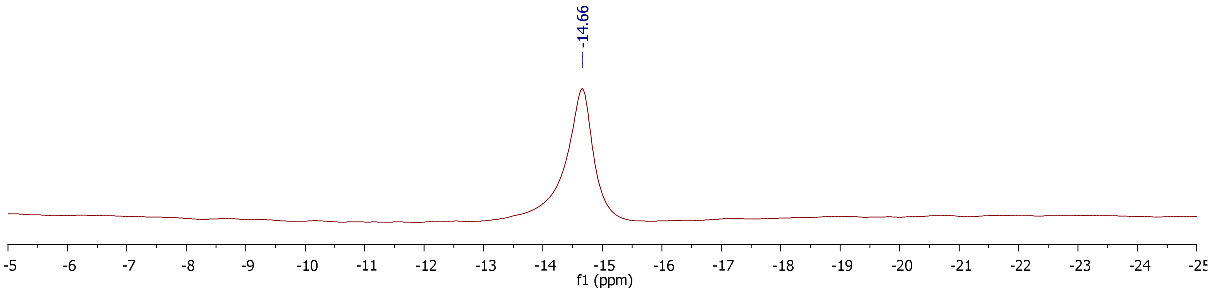

***meso*-(4-*n*-Pentylphenyl)SubTBDAP-OPh (21)**

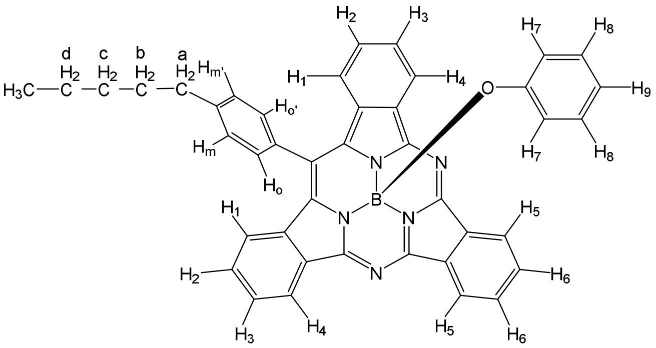

Rf 0.53 Ethyl Acetate/ Cyclohexane (2:7)

Mp 274 °C

^{1}H-NMR (500.1 MHz, CD_2Cl_2, 263 K): δ (ppm) = 8.93 – 8.89 (m, 2H, H_5), 8.89 (d, 2H, *J* = 8.0 Hz, H_4), 8.25 (d, 1H, *J* = 7.6 Hz, $H_{o'}$), 8.01 – 7.95 (m, 2H, H_6), 7.81 – 7.75 (m, 3H, $H_{m'}$, H_3), 7.59 (ddd, 2H, *J* = 8.0, 7.0, 1.0 Hz, H_2), 7.52 (br d, 2H, *J* = 8.0 Hz, H_1), 7.36 (d, 1H, *J* = 7.6 Hz, H_m), 6.89 (d, 1H, *J* = 7.6 Hz, H_o), 6.70 (t, 2H, *J* = 7.4 Hz, H_8), 6.62 (t, 1H, *J* = 7.4 Hz, H_9), 5.24 (d, 2H, , *J* = 7.4 Hz, H_7), 2.91 (t, 2H, *J* = 7.7 Hz, -C$\underline{H}_2$ a), 1.89 – 1.82 (m, 2H, -C$\underline{H}_2$ b), 1.51 – 1.41 (m, 4H, -C$\underline{H}_2$ c, d), 0.99 (t, 3H, *J* = 7.0 Hz, -C$\underline{H}_3$).

^{13}C-NMR (125.7 MHz, CD_2Cl_2, 298 K): δ (ppm) = 153.7, 150.7, 150.3, 146.7, 145.2, 134.8, 133.1, 132.8, 131.9, 130.4, 129.7, 129.1, 128.9, 128.2, 127.9, 125.3, 123.6, 122.9, 122.3, 121.8, 120.1, 36.5, 32.2, 31.8, 23.2, 14.5.

^{11}B-NMR (160.5 MHz, CD_2Cl_2, 298 K): δ (ppm) = -14.66 (s, 1B).

MS (MALDI-TOF): *m/z* = 634.34 $[M+H]^+$

UV-Vis (CH_2Cl_2): λ max (nm) (ε ($dm^3 \cdot mol^{-1} \cdot cm^{-1}$)) = 549 ($5.86 \cdot 10^4$), 327 ($3.89 \cdot 10^4$).

Fluorescence (CH_2Cl_2, Excitation at 500 nm): 559 nm, Φ_F= 0.44

^{1}H-NMR (500.1 MHz, CD_2Cl_2, 263 K)

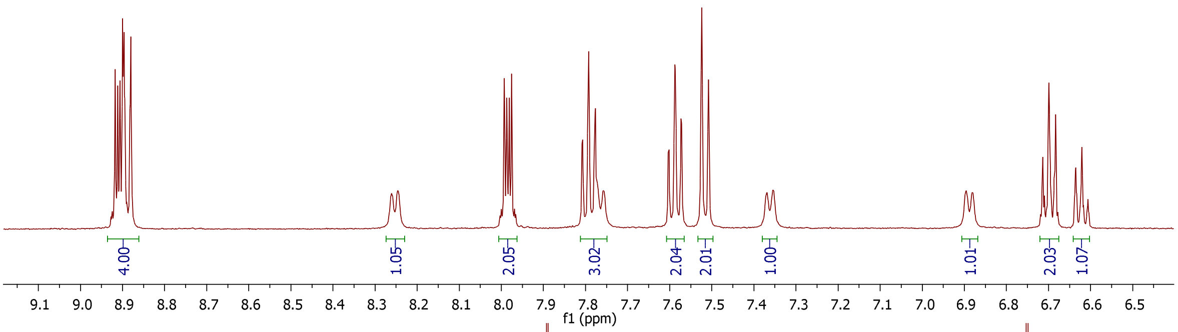

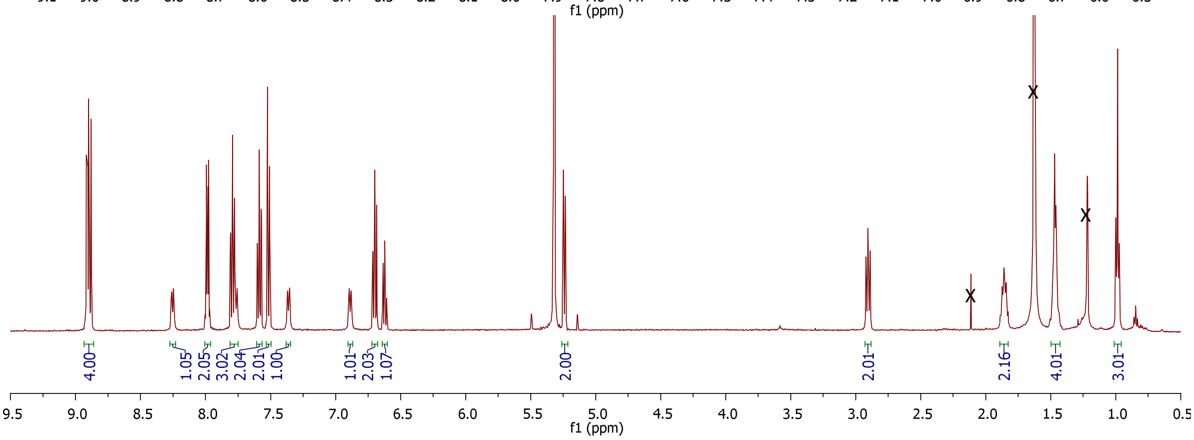

^{13}C-NMR (125.7 MHz, CD_2Cl_2, 298 K)

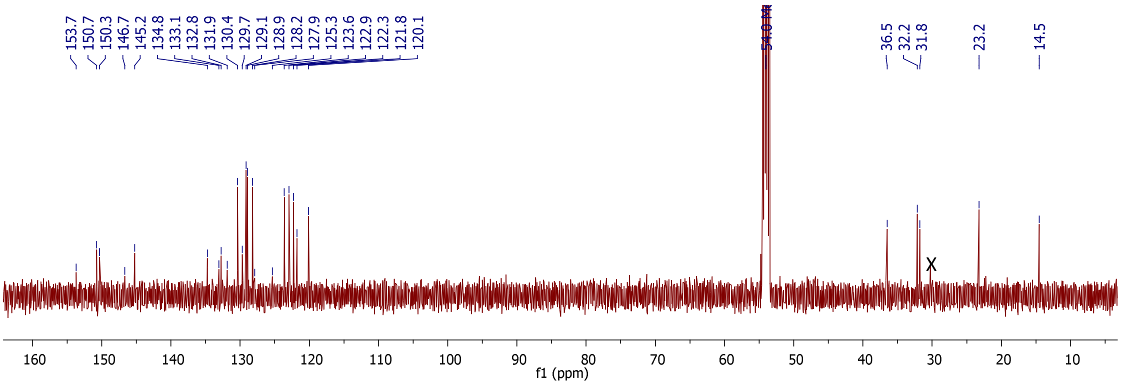

^{11}B-NMR (160.5 MHz, CD_2Cl_2, 298 K)

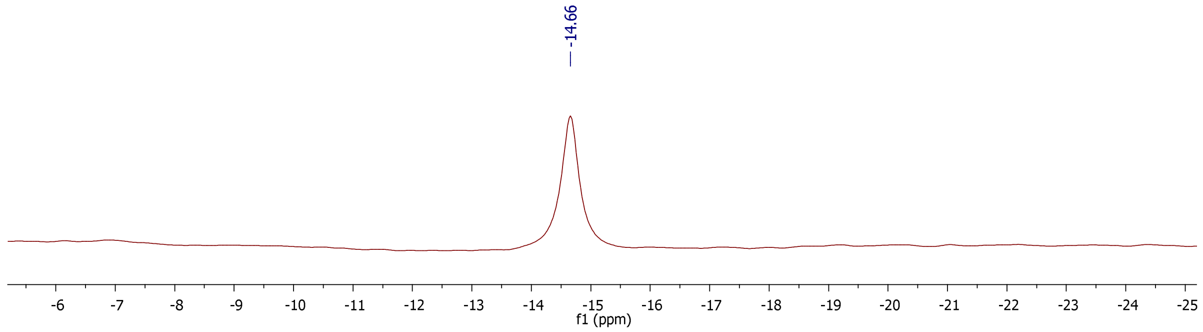

Variable temperature ROESY experiment (500.1 MHz, CD_2Cl_2, 243 K)

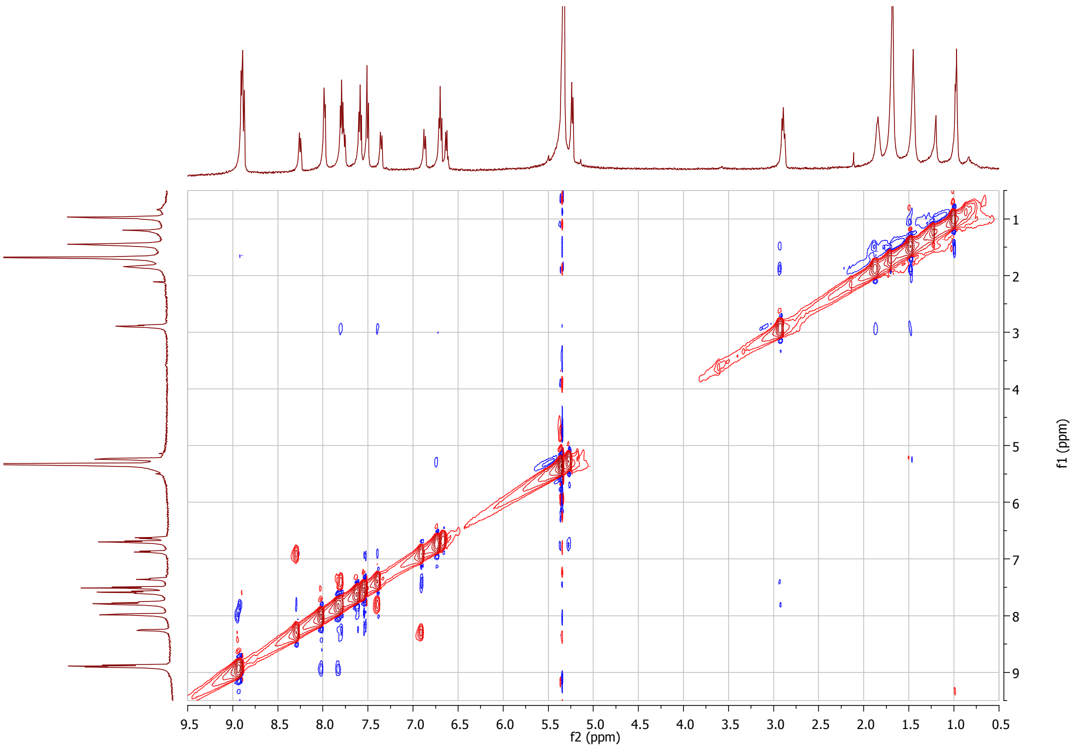

***meso*-(4-Methoxyphenyl)SubTBDAP -OPh (22)**

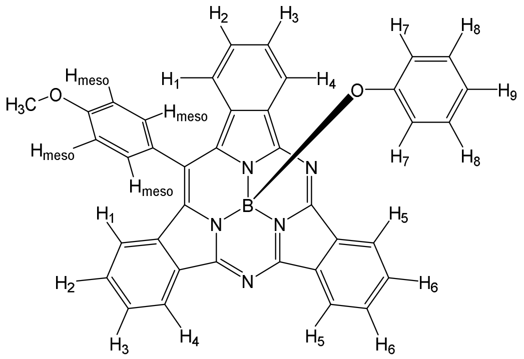

Rf 0.44 Ethyl Acetate/ Cyclohexane (2:7)

Mp 300 °C

^{1}H-NMR (500.1 MHz, CD_2Cl_2, 263 K): δ (ppm) = 8.93 – 8.89 (m, 2H, H_5), 8.89 (br d, 2H, J = 8.1 Hz, H_4), 8.28 (d, 1H, *J* = 7.6 Hz, H_{meso}), 8.01 – 7.96 (m, 2H, H_6), 7.80 (ddd, 2H, *J* = 7.9, 6.5, 1.5 Hz, H_3), 7.63 – 7.57 (m, 4H, H_2, H_1), 7.47 (d, 1H, *J* = 7.6 Hz, H_{meso}), 7.07 (d, 1H, *J* = 7.6 Hz, H_{meso}), 6.91 (d, 1H, *J* = 7.6 Hz, H_{meso}), 6.70 (t, 2H, *J* = 7.5 Hz, H_8), 6.62 (t, 2H, *J* = 7.5 Hz, H_9), 5.24 (t, 2H, *J* = 7.5 Hz, H_7), 4.05 (s, 3H, -C$\underline{H}_3$).

^{13}C-NMR (125.7 MHz, CD_2Cl_2, 298 K): δ (ppm) = 161.3, 150.7, 150.3, 134.9, 134.5, 134.0, 131.9, 130.4, 129.1, 129.0, 128.2, 127.6, 125.0, 123.6, 122.9, 122.3, 121.8, 121.3, 121.2, 120.1, 115.0, 56.1.

^{11}B-NMR (160.5 MHz, CD_2Cl_2, 298 K): δ (ppm) = -14.65 (s, 1B).

MS (MALDI-TOF): *m/z* = 593.39 $[M]^+$

HR-MS (FTMS + p NSI) ($C_{38}H_{25}B_1N_5O_2$) $[M+H]^+$: Calc.: 594.2102; Found: 594.2091.

UV-Vis (CH_2Cl_2): λ max (nm) (ε ($dm^3 \cdot mol^{-1} \cdot cm^{-1}$)) = 549 ($5.25 \cdot 10^4$), 308 ($3.41 \cdot 10^4$).

Fluorescence (CH_2Cl_2, Excitation at 500 nm): 559 nm, Φ_F= 0.45

^{1}H-NMR (500.1 MHz, CD_2Cl_2, 263 K)

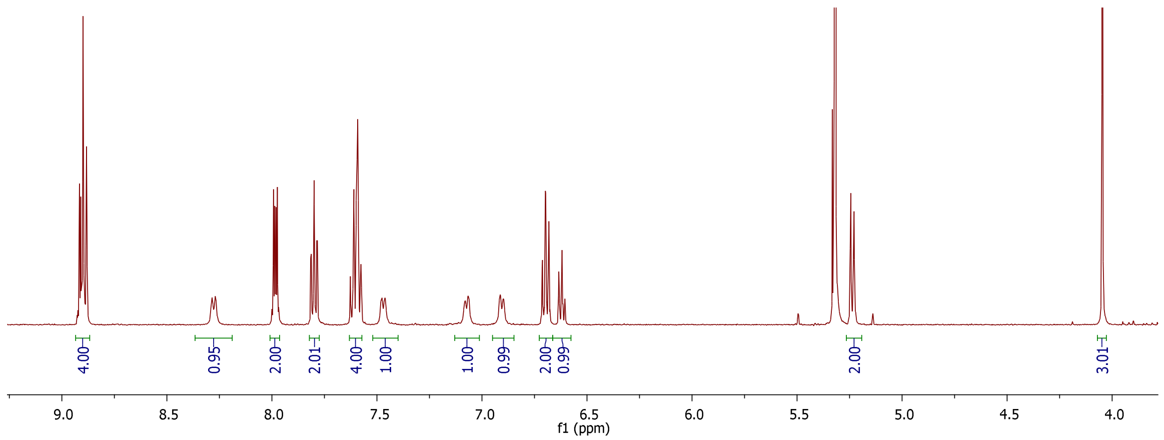

^{13}C-NMR (125.7 MHz, CD_2Cl_2, 298 K)

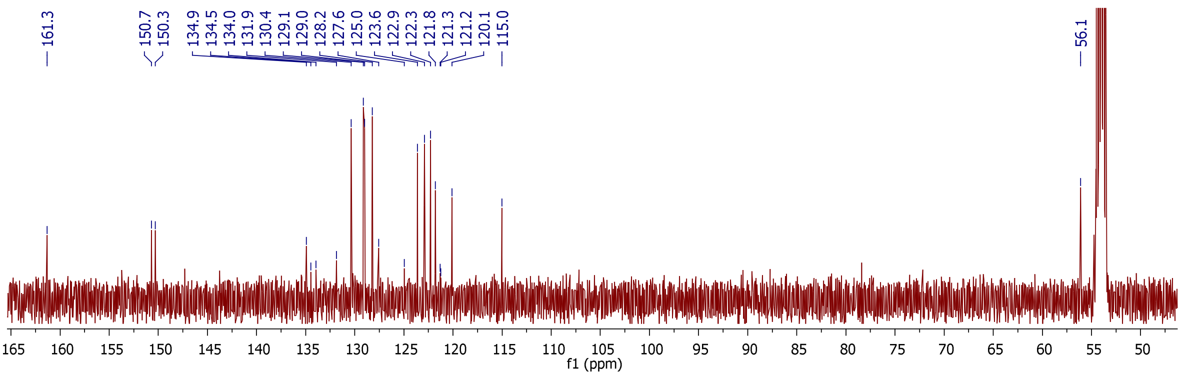

^{11}**B-NMR** (160.5 MHz, CD_2Cl_2, 298 K)

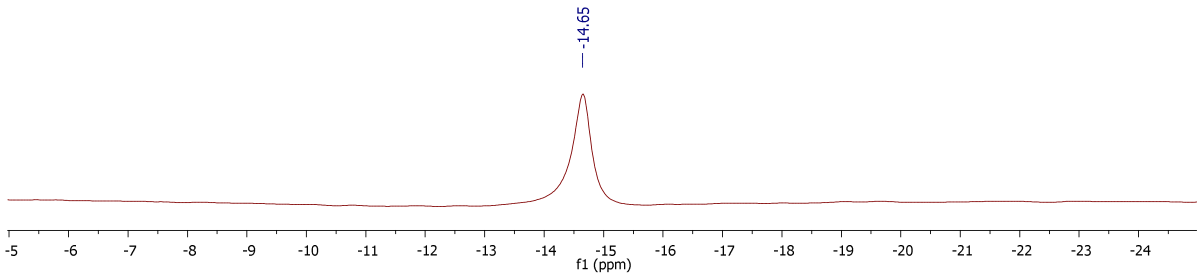

***meso*-(4-(Trifluoromethyl)phenyl)SubTBDAP-OPh (23)**

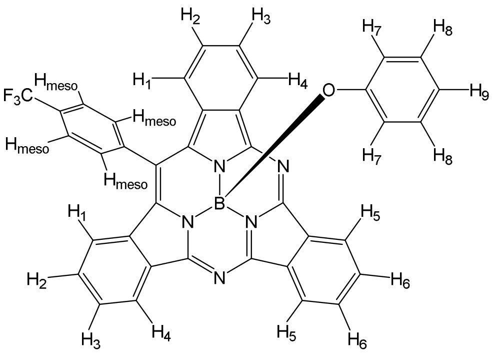

Rf 0.53 Ethyl Acetate/ Cyclohexane (2:7)

Mp 270 °C

^{1}**H-NMR** (500.1 MHz, CD_2Cl_2, 263 K): δ (ppm) = 8.96 – 8.93 (m, 4H, H_4, H_5), 8.50 (s, 1H, *J* = 7.8 Hz, H_{meso}), 8.24 (d, 1H, *J* = 7.8 Hz, H_{meso}), 8.03 –8.00 (m, 2H, H_6), 7.85 – 7.81 (m, 3H, H_{meso}, H_3), 7.62 (ddd, 2H, J = 8.1, 7.1, 1.0 Hz, H_2), 7.42 (br d, 2H, *J* = 8.1 Hz, H_1), 7.18 (d, 1H, *J* = 7.8 Hz, H_{meso}), 6.71 (t, 2H, J = 7.5 Hz, H_8), 6.64 (t, 1H, *J* = 7.5, H_9), 5.25 (d, 2H, J = 7.5 Hz H_7).

^{13}**C-NMR** (125.7 MHz, CD_2Cl_2, 298 K): δ (ppm) = 153.6, 150.8, 150.7, 139.9, 137.8, 134.2, 133.1, 132.8, 132.1 (q, 1C, ^{2}J = 32 Hz), 131.9, 130.6, 130.4, 129.3, 129.2, 128.4, 126.6 (q, 2C, ^{3}J = 4 Hz), 124.9 (q, 1C, ^{1}J = 272 Hz), 123.2, 123.0, 122.5, 121.9, 120.1.

^{11}**B-NMR** (160.5 MHz, CD_2Cl_2, 298 K): δ (ppm) = -14.67 (s, 1B).

^{19}**F-NMR** (470.6 MHz, CD_2Cl_2, 298 K): δ (ppm) = -62.64 (s, 1F, -C$\underline{F}_3$).

MS (MALDI-TOF): *m/z* = 632.15 [M+H]$^+$ (100%)

UV-Vis (CH_2Cl_2): λ max (nm) (ε ($dm^3 \cdot mol^{-1} \cdot cm^{-1}$)) = 547 ($6.72 \cdot 10^4$), 323 ($4.64 \cdot 10^4$).

Fluorescence (CH_2Cl_2, Excitation at 500 nm): 559 nm, Φ_F= 0.37

[1]**H-NMR** (500.1 MHz, CD_2Cl_2, 263 K)

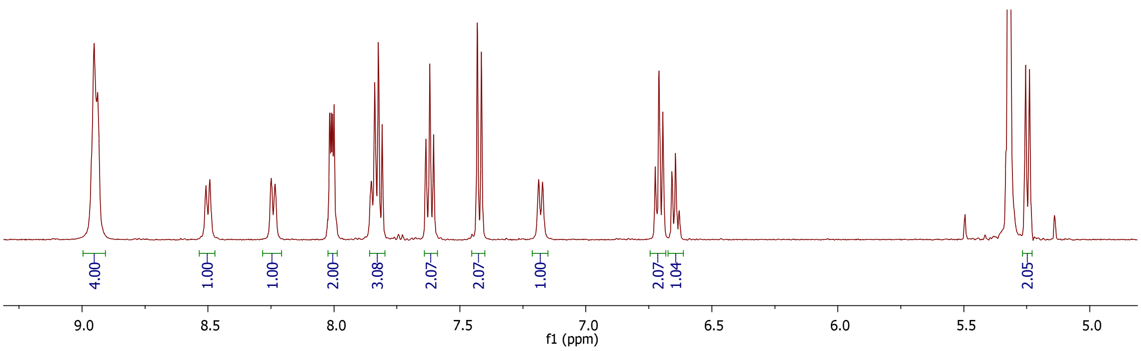

[13]**C-NMR** (125.7 MHz, CD_2Cl_2, 298 K)

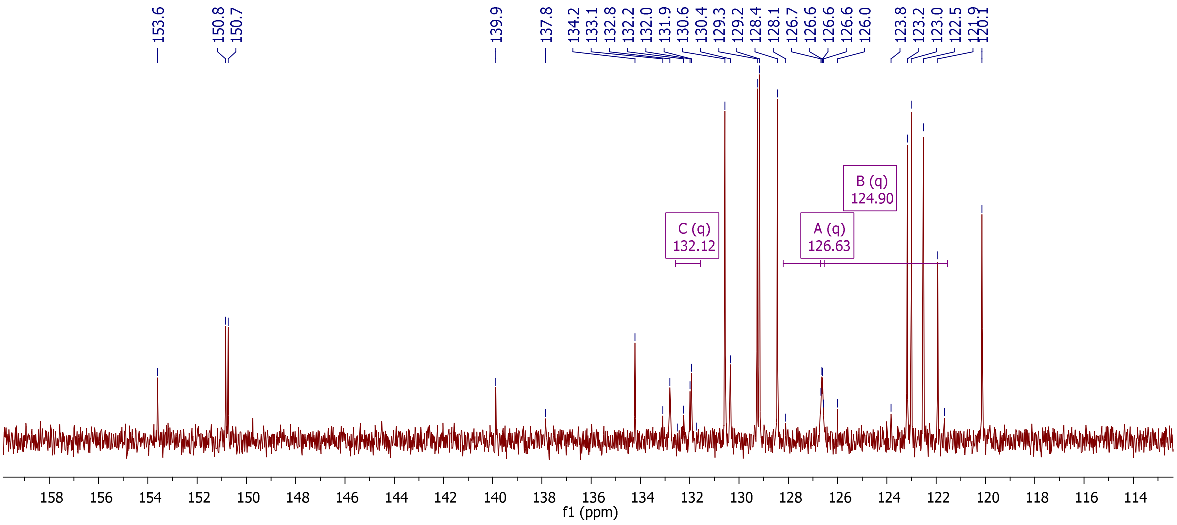

[11]**B-NMR** (160.5 MHz, CD_2Cl_2, 298 K)

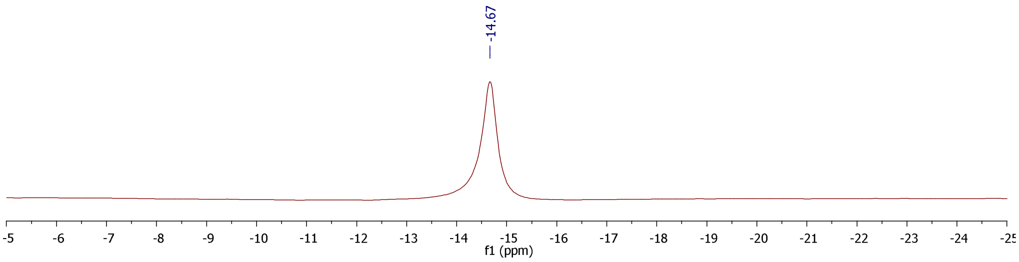

[19]**F-NMR** (470.6 MHz, CD_2Cl_2, 298 K)

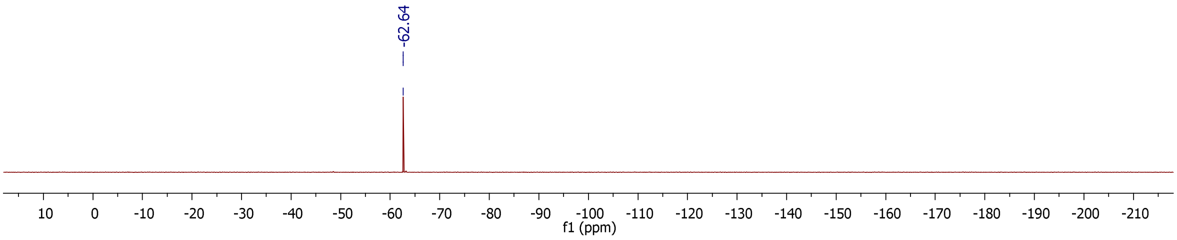

***meso*-(4-Methoxyphenyl)SubTBDAP-OMe (24)**

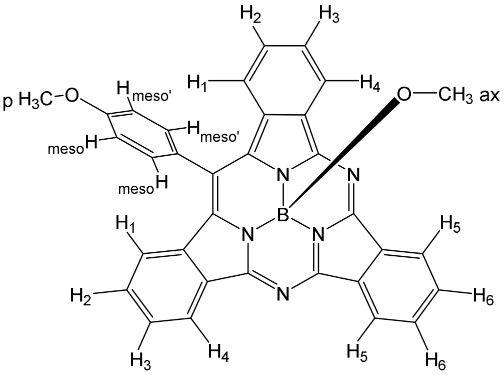

Rf 0.26 Ethyl Acetate/ Cyclohexane (2:7)

Mp 310 °C

^{1}H-NMR (500.1 MHz, CD_2Cl_2, 263 K): δ (ppm) = 8.92 – 8.87 (m, 4H, H_4, H_5), 8.48 (d, 1H, *J* = 7.6 Hz, $H_{meso'}$), 7.98 – 7.93 (m, 2H, H_6), 7.80 – 7.76 (m, 2H, H_3), 7.62 – 7.57 (m, 4H, H_1, H_2), 7.49 (d, 1H, *J* = 7.6 Hz, $H_{meso'}$), 7.07 (d, 1H, *J* = 7.6 Hz, H_{meso}), 6.88 (d, 1H, *J* = 7.6 Hz, H_{meso}), 4.05 (s, 3H, -C$\underline{H}_3$ p), 1.27 (s, 3H, -C$\underline{H}_3$ ax).

^{13}C-NMR (125.7 MHz, CD_2Cl_2, 298 K): δ (ppm) = 161.3, 151.1, 150.6, 135.2, 133.1, 131.9, 130.3, 130.2, 128.8, 128.1, 127.8, 124.8, 123.5, 122.8, 122.2, 120.4, 115.0, 56.1, 47.0.

^{11}B-NMR (160.5 MHz, $CDCl_3$, 298 K): δ (ppm) = -14.44 (s, 1B).

MS (MALDI-TOF): *m/z* = 531.26 $[M]^+$ (100%)

HR-MS (FTMS + p NSI) ($C_{33}H_{23}B_1N_5O_2$) $[M+H]^+$: Calc.: 532.1945; Found: 532.1935

UV-Vis (CH_2Cl_2): λ max (nm) (ε ($dm^3 \cdot mol^{-1} \cdot cm^{-1}$)) = 548 ($5.48 \cdot 10^4$), 322 ($3.49 \cdot 10^4$).

Fluorescence (CH_2Cl_2, Excitation at 500 nm): 558 nm, Φ_F= 0.44.

^{1}H-NMR (500.1 MHz, CD_2Cl_2, 263 K)

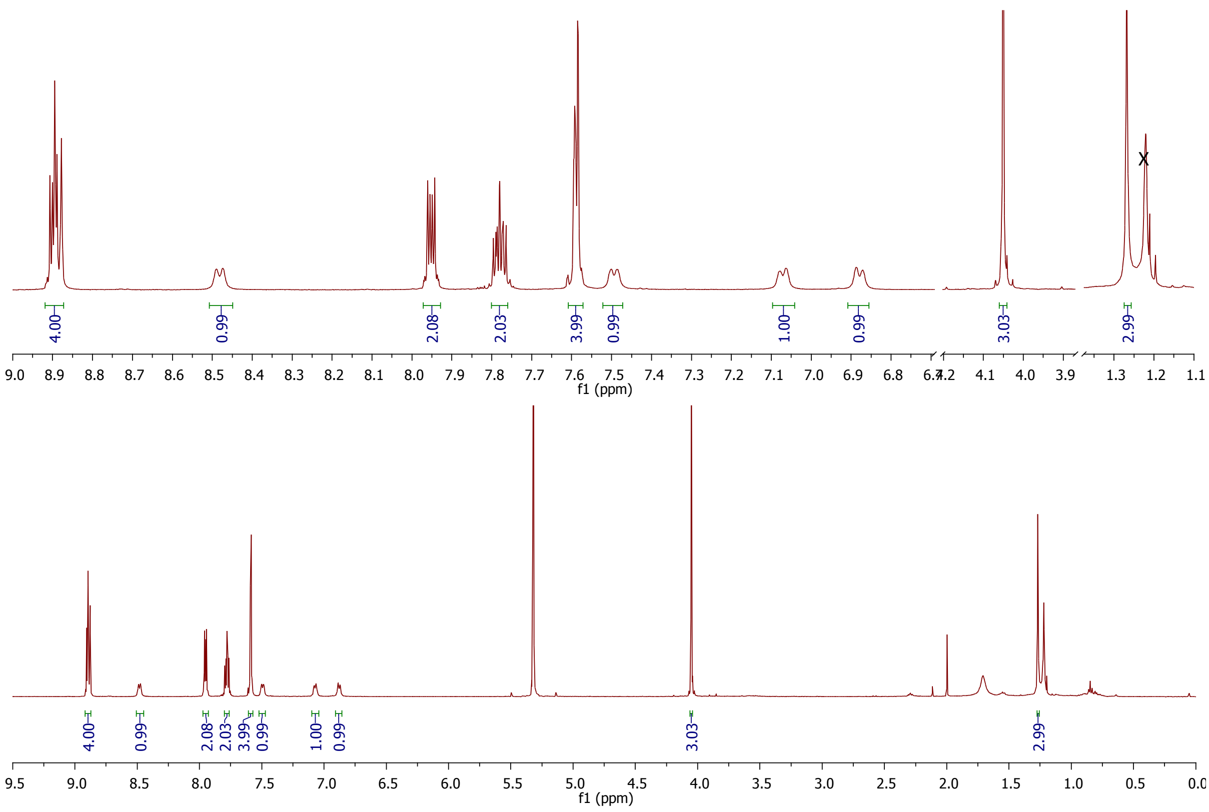

^{13}C-NMR (125.7 MHz, CD_2Cl_2, 298 K)

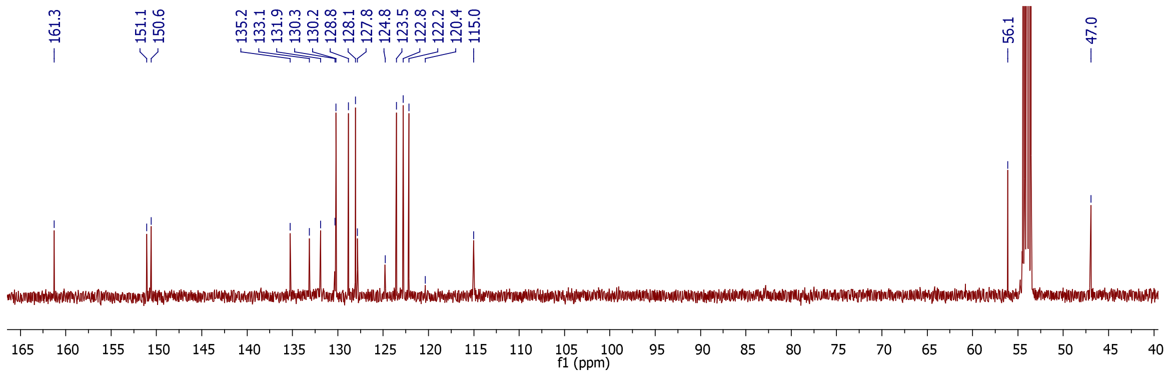

^{11}B-NMR (160.5 MHz, $CDCl_3$, 298 K)

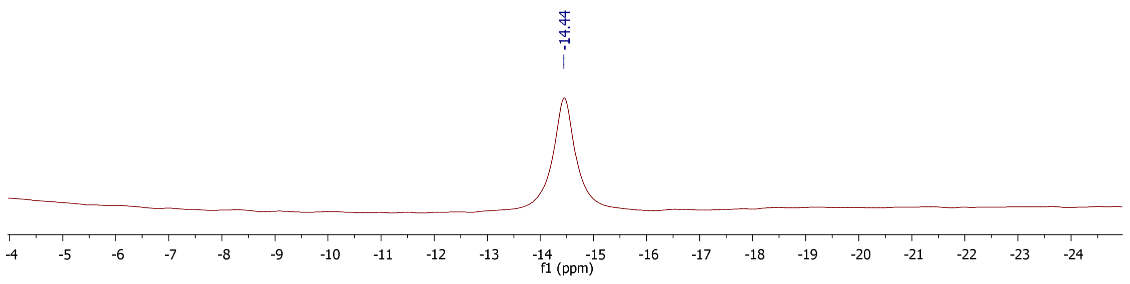

VT COSY experiment (500.1 MHz, CD_2Cl_2, 263 K)

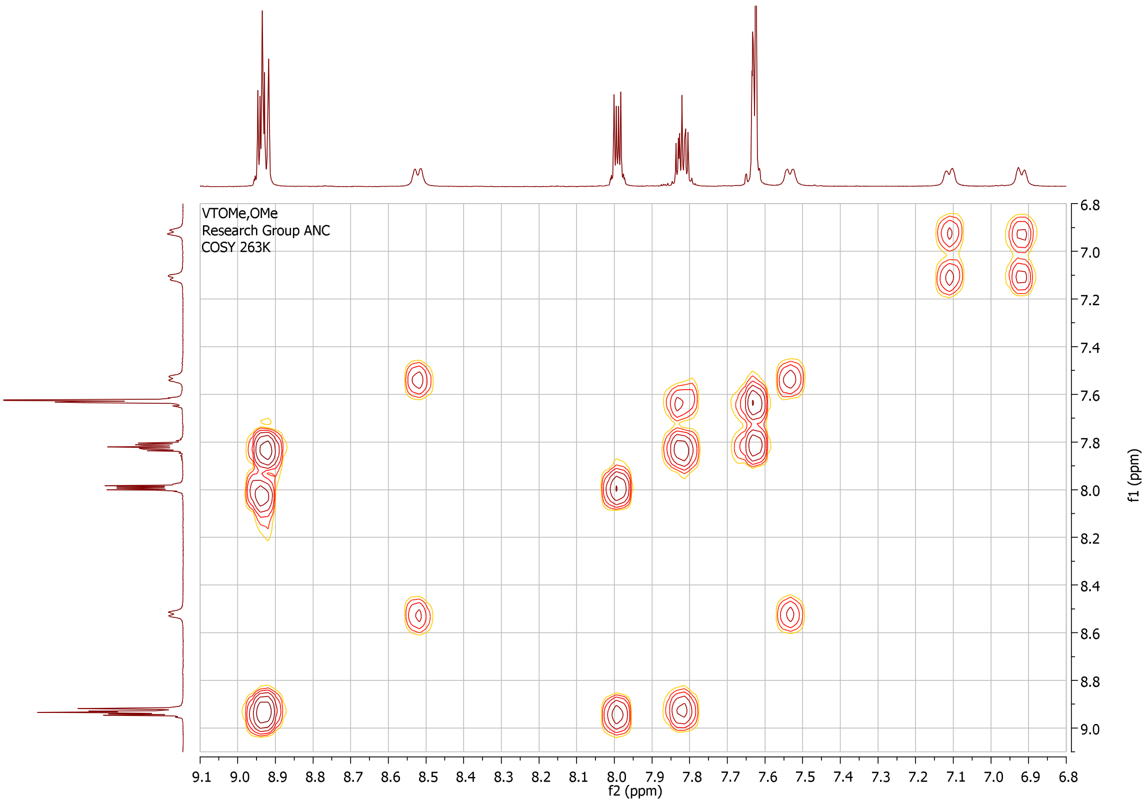

[1] R. Ahlrichs, M. Bär, M. Häser, H. Horn, C. Kölmel, Chem. Phys. Lett., 1989, 162, 165-169. b) J. P. Perdew, Phys. Rev B., 1986, 33, 8822-8824.

[2] A. D. Becke, Phys. Rev. A, 1988, 38, 3098-3100.

[3] R. Velapoldi, H. Tønnesen, *Journal of Fluorescence* **2004**, *14*, 465-472.

[4] M. Hellal, G. D. Cuny, *Tetrahedron Lett.* **2011**, *52*, 5508-5511.

[5] A. Díaz-Moscoso, E. Emond, D. L. Hughes, G. J. Tizzard, S. J. Coles, A. N. Cammidge, *The Journal of Organic Chemistry* **2014**, *79*, 8932-8936.

[6] A. Díaz-Moscoso, G. J. Tizzard, S. J. Coles, A. N. Cammidge, *Angew. Chem. Int. Ed.* **2013**, *52*, 10784-10787.

[7] S. Dalai, V. N. Belov, S. Nizamov, K. Rauch, D. Finsinger, A. de Meijere, *Eur. J. Org. Chem.* **2006**, 2753-2765.

X-Ray Data obtained at UEA

***meso*-PhenylSubTBDAP-OPh (13)**

A single crystal suitable for X-ray analysis was prepared by recrystallization from a mixture of acetone/hexane.

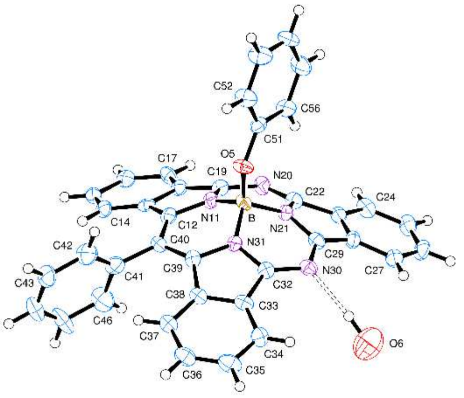

Crystal data: $C_{37}H_{22}BN_5O$, H_2O, M = 581.42. Monoclinic, space group $P2_1/n$ (equiv. to no. 14), a = 11.0275(5), b = 13.7317(8), c = 17.8943(8) Å, β = 91.644(4) °, V = 2708.6(2) Å^3. Z = 4, Dc = 1.426 g cm^{-3}, F(000) = 1208, T = 140(1) K, μ(Mo-Kα) = 0.09 cm^{-1}, λ(Mo-Kα) = 0.71069 Å.
Crystals are red prisms. From a sample under oil, one, *ca* 0.05 x 0.10 x 0.41 mm, was mounted on a glass fibre and fixed in the cold nitrogen stream on an Oxford Diffraction Xcalibur-3/Sapphire3-CCD diffractometer, equipped with Mo-Kα radiation and graphite monochromator. Intensity data were measured by thin-slice ω- and ϕ-scans. Total no. of reflections recorded, to θ_{max} = 21.5°, was 24461 of which 3107 were unique (Rint = 0.111); 2074 were 'observed' with $I > 2\sigma_I$.
Data were processed using the CrysAlisPro-CCD and -RED (1) programs. The structure was determined by the direct methods routines in the SHELXS program (2A) and refined by full-matrix least-squares methods, on F^2's, in SHELXL (2B). The non-hydrogen atoms were refined with anisotropic thermal parameters. Hydrogen atoms in the main molecule were included in idealised positions and their Uiso values were set to ride on the Ueq values of the parent carbon atoms. There were a number of persistent difference peaks in the neighbourhood of the solvent water oxygen atom, O(6), and four of these were included as hydrogen atoms which were refined freely; two appear close to likely sites as part of the water molecule, whilst two were further from the oxygen atom and might be indications of site disorder of the solvent molecule. At the conclusion of the refinement, wR_2 = 0.140 and R_1 = 0.098 (2B) for all 3107 reflections weighted w = $[\sigma^2(F_o^2) + (0.0631P)^2]^{-1}$ with P = $(F_o^2 + 2F_c^2)/3$; for the 'observed' data only, R_1 = 0.057.
In the final difference map, the highest peak (*ca* 0.23 eÅ^{-3}) was near H(37) .
Scattering factors for neutral atoms were taken from reference (3). Computer programs used in this analysis have been noted above, and were run through WinGX (4) on a Dell Optiplex GX620 PC at the University of East Anglia.

References

(1) Programs CrysAlisPro, Oxford Diffraction Ltd., Abingdon, UK (2010).

(2) G. M. Sheldrick, SHELX-97 – Programs for crystal structure determination (SHELXS) and refinement (SHELXL), *Acta Cryst.* (2008) A64, 112-122.

(3) *'International Tables for X-ray Crystallography'*, Kluwer Academic Publishers, Dordrecht (1992). Vol. C, pp. 500, 219 and 193.

(4) L. J. Farrugia, (2012) *J. Appl. Cryst.* 45, 849–854.

X-Ray Data obtained at Southampton

meso-PhenylSubTBDAP-O^{i}Pr (15)

A single crystal suitable for X-Ray analysis was grown from a mixture of cyclohexane/acetone.

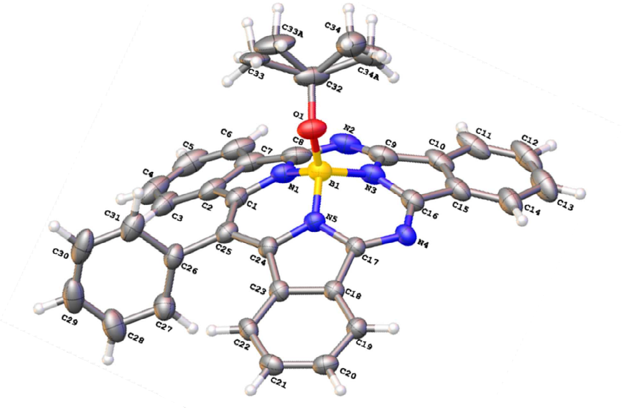

Formula: $C_{34}H_{24}BN_5O$; M_r = 529.39; crystal dimensions: 0.09 x 0.08 x 0.08 mm; crystal system: Monoclinic; space group: *P2₁/c*; a = 9.391(2) Å, b = 28.565(6) Å, c = 10.538(3) Å, α = 90°, β = 113.175(4)°, γ = 90°; V = 2598.8(11) Å^3; Z = 4; ρ_{calcd} = 1.353 Mg/m^3; μ = 0.084 mm^{-1}; Mo $K\alpha$ radiation, λ = 0.71075 Å; T = 100 K; $2\Theta_{max}$ = 50°; 27657/4574 measured/independent reflections; R_{int}: 0.0808; $R1[F^2 > 2\sigma(F^2)]$ = 0.0514, $wR2$(all data) = 0.1281; $\Delta\rho_{max}$ = 0.288 eÅ^{-3}, $\Delta\rho_{min}$ = −0.193 eÅ^{-3}. Red block crystals were poorly diffracting with no significant data beyond 0.84Å. The data were collected on a Rigaku Saturn 724+ area detector mounted at the window of an FR-E+ rotating anode generator with a Mo anode and equipped with an Oxford Cryosystems cryostream device. Rigaku CrystalClear[1] was used to record images and for data integration. The structure was solved by charge-flipping methods using SUPERFLIP[2] and refined on F_o^2 by full-matrix least squares refinement using SHELXL-2014.[3] All non-hydrogen atoms were refined with anisotropic displacement parameters. Hydrogen atoms were added at calculated positions and refined using a riding model with isotropic displacement parameters based on the equivalent isotropic displacement parameter (Ueq) of the parent atom. The isopropoxide substituent is disordered over two positions (60:40). The structure was deposited on the Cambridge Structural Database with the deposition number CCDC 1052412.

***meso*-PhenylSubTBDAP-OMe (16)**

Crystals suitable for X-Ray analysis were grown dissolving SubTBDAP **16** in a mixture of dichloromethane/hexane and allowing slow diffusion with acetone.

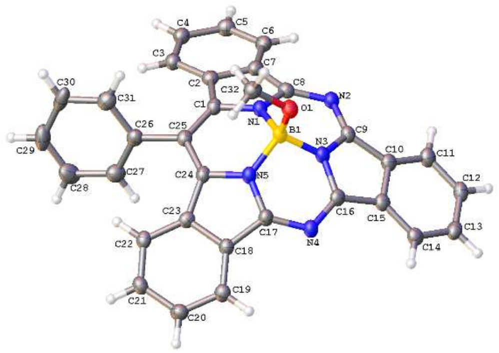

Formula: $C_{32}H_{20}BN_5O$; M_r = 501.35; crystal dimensions: 0.11 x 0.06 x 0.01 mm; crystal system: Monoclinic; space group: *P2₁/c*; a = 11.856(2) Å, b = 8.8935(17) Å, c = 21.923(4) Å, α = 90°, β = 92.213(3)°, γ = 90°; V = 2309.9(7) Å^3; Z = 4; ρ_{calcd} = 1.442 Mg/m^3; μ = 0.090 mm^{-1}; Mo $K\alpha$ radiation, λ = 0.71075 Å; T = 100 K; $2\Theta_{max}$ = 55°; 15778/5289 measured/independent reflections; R_{int}: 0.0601; $R1[F^2 > 2\sigma(F^2)]$ = 0.0605, $wR2$(all data) = 0.1675; $\Delta\rho_{max}$ = 1.097 eÅ^{-3}, $\Delta\rho_{min}$ = −0.224 eÅ^{-3}. Red plate crystals gave good diffraction. The data were collected on a Rigaku Saturn 724+ area detector mounted at the window of an FR-E+ rotating anode generator with a Mo anode and equipped with an Oxford Cryosystems cryostream device. Rigaku CrystalClear[1] was used to record images and for data integration. The structure was solved by charge-flipping methods using SUPERFLIP[2] and refined on F_o^2 by full-matrix least squares refinement using SHELXL-2013.[3] All non-hydrogen atoms were refined with anisotropic displacement parameters. Hydrogen atoms were added at calculated positions and refined using a riding model with isotropic displacement parameters based on the equivalent isotropic displacement parameter (Ueq) of the parent atom. The structure was deposited on the Cambridge Structural Database with the deposition number CCDC 1044583.

***meso*-PhenylSubTBDAP-OBu (17)**

Crystals suitable for X-Ray were grown dissolving SubTBDAP **17** in a mixture of dichloromethane/acetone allowing slow diffusion of cyclohexane.

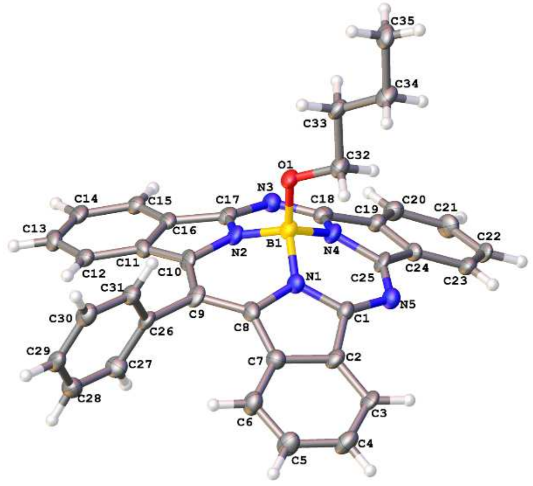

Formula: $C_{35}H_{26}BN_5O$; M_r = 543.42; crystal dimensions: 0.07 x 0.04 x 0.01 mm; crystal system: Monoclinic; space group: $P2_1/c$; a = 9.6971(8) Å, b = 30.054(3) Å, c = 9.8109(8) Å, α = 90°, β = 110.468(9)°, γ = 90°; V = 2678.8(4) Å^3; Z = 4; ρ_{calcd} = 1.347 Mg/m^3; μ = 0.083 mm^{-1}; Mo $K\alpha$ radiation, λ = 0.71075 Å; T = 100 K; $2\Theta_{max}$ = 50°; 19159/4732 measured/independent reflections; R_{int}: 0.1086; $R1[F^2 > 2\sigma(F^2)]$ = 0.0936, $wR2$(all data) = 0.1924; $\Delta\rho_{max}$ = 0.313 eÅ^{-3}, $\Delta\rho_{min}$ = −0.337 eÅ^{-3}. Red plate crystals were poorly diffracting with no significant data beyond 0.84Å. The data were collected on a Rigaku Saturn 724+ area detector mounted at the window of an FR-E+ rotating anode generator with a Mo anode and equipped with an Oxford Cryosystems cryostream device. Rigaku CrystalClear[1] was used to record images and Agilent CrysAlisPro[4] was used for data integration. The structure was solved by charge-flipping methods using SUPERFLIP[2] and refined on F_o^2 by full-matrix least squares refinement using SHELXL-2014.[3] All non-hydrogen atoms were refined with anisotropic displacement parameters. Hydrogen atoms were added at calculated positions and refined using a riding model with isotropic displacement parameters based on the equivalent isotropic displacement parameter (Ueq) of the parent atom. The structure was deposited on the Cambridge Structural Database with the deposition number CCDC 1044584.

meso-(4-Methoxyphenyl)SubTBDAP-OMe (24)

Crystals suitable for X-Ray analysis were grown by dissolving SubTBDAP **24** in a mixture of dichloromethane/acetone and allowing slow diffusion of methanol.

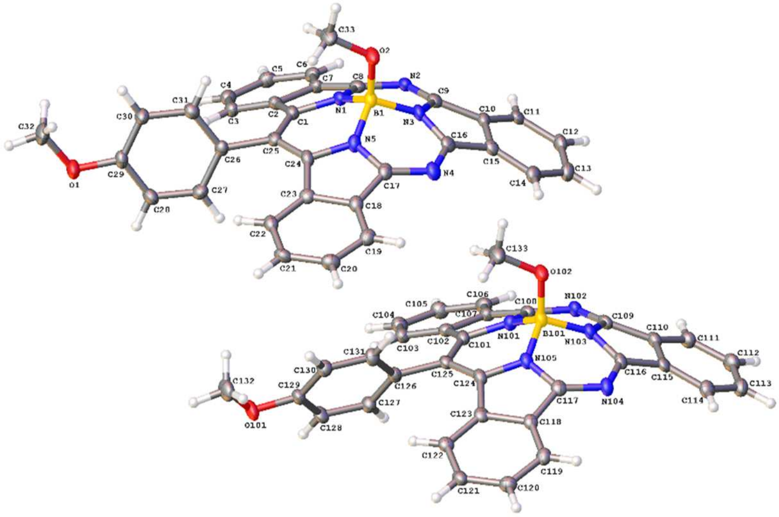

Formula: $C_{33}H_{22}BN_5O_2$; M_r = 531.36; crystal dimensions: 0.06 x 0.06 x 0.01 mm; crystal system: Monoclinic; space group: $P2_1/n$; a = 15.0996(6) Å, b = 17.6493(8) Å, c = 18.9194(9) Å, α = 90°, β = 96.370(4)°, γ = 90°; V = 5010.9(4) Å^3; Z = 8; ρ_{calcd} = 1.409 Mg/m^3; μ = 0.090 mm^{-1}; Mo Kα radiation, λ = 0.71075 Å; T = 100 K; 2Θmax = 55°; 62368/11493 measured/independent reflections; R_{int}: 0.1519; $R1[F2 > 2\sigma(F^2)]$ = 0.0651, $wR2$(all data) = 0.1445; $\Delta\rho_{max}$ = 0.271 eÅ^{-3}, $\Delta\rho_{min}$ = −0.271 eÅ^{-3}. Red plate crystals gave good diffraction. The data were collected on a Rigaku Saturn 724+ area detector mounted at the window of an FR-E+ rotating anode generator with a Mo anode and equipped with an Oxford Cryosystems cryostream device. Rigaku CrystalClear[1] was used to record images and Agilent CrysAlisPro[4] was used for data integration. The structure was solved by charge-flipping methods using SUPERFLIP[2] and refined on F_o^2 by full-matrix least squares refinement using SHELXL-2014.[3] All non-hydrogen atoms were refined with anisotropic displacement parameters. Hydrogen atoms were added at calculated positions and refined using a riding model with isotropic displacement parameters based on the equivalent isotropic displacement parameter (Ueq) of the parent atom. The structure was deposited on the Cambridge Structural Database with the deposition number CCDC 1044585.

References

[1] CrystalClear, 2008-2013, Rigaku Corporation, The Woodlands, Texas, U.S.A.

[2] L. Palatinus, G. Chapuis, J. Appl. Cryst. 2007, 40, 786-790.

[3] G. M. Sheldrick, Acta Cryst. A, 2008, A64, 112-122.

[4] CrysAlisPro Software system, version 1.171.37.31, 2014, Agilent Technologies UK Ltd, Oxford, UK
